# Trained Immunity and Cardiovascular Risk: An Immunological Perspective

**DOI:** 10.1111/imr.70095

**Published:** 2026-01-05

**Authors:** Katherine A. Boden, Jason Chai, Tafadzwa T. J. Kufazvinei, Robin P. Choudhury

**Affiliations:** ^1^ Division of Cardiovascular Medicine, Radcliffe Department of Medicine University of Oxford Oxford UK

**Keywords:** cardiovascular disease, inflammation, trained immunity

## Abstract

Systemic inflammation is a key driver of atherogenesis and its complications. While anti‐inflammatory therapies targeting pathways such as IL‐1β and IL‐6 have shown promise in established atherosclerotic cardiovascular disease (ASCVD), potential systemic effects raise concerns about immune suppression and infection, underscoring the need for more precise immunomodulatory approaches. Trained immunity—a form of innate immune memory—has emerged as a potential contributor linking ASCVD risk factors to chronic inflammation and disease progression. In this review, we discuss the evidence for trained immunity in ASCVD, its induction by several known risk factors (e.g., hyperglycaemia, hypercholesterolemia, diet, chronic stress, inflammatory diseases, and infection), and its potential role in sustaining vascular inflammation. Advancing our understanding of the metabolic and epigenetic mechanisms underlying trained immunity, as well as defining shared and cumulative effects across risk factors, will be critical to guide the development of next‐generation targeted therapies for ASCVD prevention and treatment.

## 1 Introduction

The involvement of inflammation in cardiovascular disease was first recognized more than three decades ago, when systemic inflammatory markers (specifically the acute‐phase proteins C‐reactive protein (CRP) and serum amyloid A) were associated with adverse outcomes, initially in patients with acute coronary syndromes [1] and subsequently in apparently healthy men [2]. More recently, in a collaborative analysis of three randomized clinical trials, Ridker and colleagues showed that in patients in whom LDL‐cholesterol was treated to contemporary standards, inflammation assessed by high‐sensitivity C‐reactive protein (hsCRP) was a stronger predictor for risk of future cardiovascular events and death than on‐treatment LDL cholesterol [3]. In line with this, clinical studies have shown that increased CRP and proinflammatory remodeling of circulating leukocytes in patients with acute coronary syndrome (ACS) predicts the likelihood of recurrence of cardiovascular events [4]. These data fall into a broader landscape that identifies a role of inflammation as a target for reducing risk in cardiovascular disease.

Several recent randomized controlled trials demonstrate that targeted immunomodulation can reduce cardiovascular event risk. However, enthusiasm for a widespread application of imprecisely targeted “anti‐inflammatory” drugs has been tempered by concerns around potential infection risk and the high financial cost of using expensive biologicals in populations that are not identified based on known/high probability mechanistic susceptibility to treatment.

At population level, hsCRP, an acute phase protein made by the liver, has repeatedly been shown to predict cardiovascular risk. However, while hsCRP serves as an integrated indicator of systemic infection it (a) gives little indication of the particular underlying processes of inflammation and (b) is not itself on the causal pathway for atherosclerotic cardiovascular disease (ASCVD) [5].

To capitalize on translational opportunities to target pathogenic processes of inflammation means to characterize these processes in individual patients, with a view to guiding therapies targeting inflammation reprogramming and immunomodulation that are urgently needed. Accumulating evidence implicates innate immune memory, termed “trained immunity”, as a central mechanism driving pathogenic inflammation and impeding homeostatic repair in ASCVD. After exposure to environmental risk factors or pathogens, monocytes can undergo persistent changes in immune cell function that alter both cellular metabolism (favoring aerobic glycolysis), epigenetic status, and promote processes of inflammation while impairing pathways linked to repair and remediation [6]. The potential significance of trained immunity in ASCVD is highlighted by its presence in patients with established symptomatic atherosclerosis [7], acute myocardial infarction (AMI) [8], and now also unstable angina with high hsCRP [9].

In this review, we will critically evaluate the relationship between established drivers of ASCVD and trained immunity. We will consider how multiple inflammatory drivers may converge through epigenetic reprogramming of bone marrow stem cells, fundamentally altering the metabolic profile and the inflammatory or homeostatic functions of their progeny. We will also discuss how these changes can persist long‐term, be detected in peripheral cells, and form the basis for new approaches to both individualized diagnostic tools and targeted therapeutic interventions. These considerations are timely and highlight new opportunities for the prevention and treatment of ASCVD.

## 2 Trained Immunity Background

The phenomenon of ‘trained immunity’, first described in 2011, is an ability of innate immune cells to acquire a persistent, non‐specific immunological memory of prior functionally important exposures, which results in an enhanced or modified response to subsequent immune stimuli [10]. This concept was demonstrated in humans in 2012 [11], when Bacillus Calmette–Guérin (BCG) vaccination in healthy volunteers was shown to lead to the functional reprogramming of monocytes, such that they elicited an enhanced pro‐inflammatory response to secondary challenge with unrelated pathogens.

Immunologically induced trained immunity develops after pathogen‐associated molecular patterns (PAMPs), such as β‐glucan and BCG, bind to cells of the innate immune system via pattern recognition receptors (PRRs) expressed on their cell surface and cytoplasm. This leads to changes in cellular signaling and metabolism that culminate in long‐term metabolic and epigenetic reprogramming (detailed below). This reprogramming, in turn, renders cells “primed” for enhanced secretion of pro‐inflammatory cytokines (e.g., tumor necrosis factor alpha (TNF‐α), interleukin 6 (IL‐6), and IL‐1β) and chemokines (e.g., C‐X‐C Motif Chemokine Ligand (CXCL)‐9–11) upon secondary stimulation.

### 2.1 Metabolic Reprogramming

At a molecular level, immune stimulation results in the convergence of multiple regulatory pathways, including changes in cellular metabolism (e.g., glycolysis, glutaminolysis, mevalonate pathway, the tricarboxylic acid (TCA) cycle, arginine‐derived metabolites, and oxidative phosphorylation) essential to the establishment of functional trained immunity programmes in innate immune cells and their progenitors. Specifically, trained immunity is associated with a metabolic shift of central glucose metabolism from oxidative phosphorylation to aerobic glycolysis (similar to the “Warburg effect”), regulated via mechanistic target of rapamycin (mTOR), which stabilizes and increases translation of hypoxia inducible factor 1α (HIF‐1α), and in turn, HIF‐1α promotes transcription of glycolytic enzymes and glucose transporters (e.g., GLUT1, hexokinase, phosphofructokinase), thereby enhancing glycolytic flux while suppressing reliance on mitochondrial oxidative phosphorylation [12]. This metabolic reprogramming results in increased glucose consumption and high lactate accumulation. Many trained immunity stimuli converge on glycolysis and, importantly, the inhibition of glycolysis abolishes the trained immunity phenotype in some models [9, 12, 13, 14, 15, 16], demonstrating the significance of glycolysis as a key metabolic pathway in the formation of innate immune cell memory. In line with this, associations between single nucleotide polymorphisms (SNPs) in key glycolytic genes, including 6‐phosphofructo‐2‐kinase/fructose‐2,6‐biphosphatase 3 (PFKFB3) and phosphofructokinase (PFKP), have been found to influence the training capacity of monocytes isolated from healthy volunteers [14]. Moreover, a recent study reports that trained monocytes preferentially employ lactate as a TCA cycle substrate, and lactate metabolism is required for trained immune cell responses to bacterial and fungal infection [17]. According to this model, a portion of accumulated lactate serves as a primary source of acetyl‐CoA production for the TCA cycle in trained monocytes. The remaining lactate enters the nucleus and binds the loci of cytokine promoters (histone lactylation, discussed in detail below), leading to and maintaining the accessibility of chromatin at cytokine gene loci [17]. Inhibiting lactate‐dependent metabolism by silencing lactate dehydrogenase A (LDHA) impaired both lactate‐fuelled TCA cycle and histone lactylation [17].

Although we and others have focused on the role of glycolysis, there are simultaneous and important changes in other metabolic pathways and metabolites that contribute to the development of trained immunity. For example, glutaminolysis (the conversion of glutamine into glutamate) also plays a central role in the induction of trained immunity. Specifically, increased rates of glutaminolysis replenish TCA cycle intermediates, resulting in the accumulation of succinate, fumarate, and malate [18, 19]. The central role of fumarate has been exemplified by its ability to dose‐dependently induce training in monocytes in vitro [18]. Mechanistically, fumarate accumulation contributes to the trained immunity phenotype in at least two ways: (i) by inhibiting KDM5 histone lysine demethylases and thus influencing monocyte epigenetic reprogramming [18] and (ii) through inhibition of HIF‐1α proteasomal degradation, which maintains the increased glycolytic flux necessary for trained immunity [20].

In addition to the role of fumarate, excess acetyl‐CoA (generated via a truncated TCA cycle where pyruvate is converted into acetyl‐CoA and citrate and then exported to the cytosol to be converted back into acetyl‐CoA by ATP citrate lyase) enters the mevalonate pathway. This leads to accumulation of the metabolite mevalonate, which dose‐dependently induces trained immunity in vitro via the activation of insulin growth factor‐1 receptor (IGF‐1R)–mTOR signaling [15]. This creates a positive‐feedback loop whereby glycolysis promotes mevalonate accumulation, which subsequently amplifies glycolysis via IGF‐1R signaling. In support of the role of mevalonate, monocytes from patients with a mevalonate kinase deficiency, termed hyper immunoglobulin D syndrome (HIDS), accumulate mevalonate and have a constitutive trained immunity phenotype at both the immunological and epigenetic levels [15].

Furthermore, arginine and its metabolites have also been reported as involved in the induction of trained immunity [21]. Arginine deprivation or arginase inhibition during β‐glucan training impaired the amplification of IL‐6 and TNF cytokine response to LPS, and genetic studies revealed polymorphisms near genes coding for arginine‐metabolizing enzymes modulated the induction of trained immunity [21]. Indeed, arginine starvation of T cells has been found to result in a genome‐wide reduction in chromatin accessibility and increased repressive H3K27me3 modification, which was associated with the inability of these cells to fully activate [22]. It is possible that a link between amino acid deprivation and changes in epigenetic programming exists in innate immune cells during trained immunity.

### 2.2 Epigenetic Reprogramming

**FIGURE 1:**
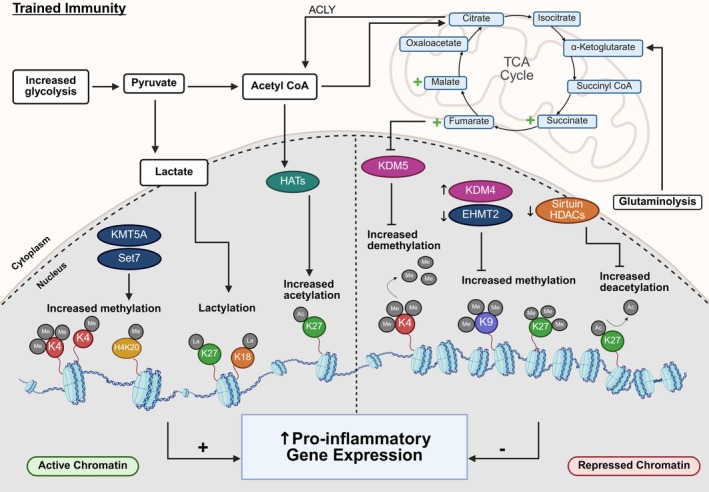
**Metabolic‐epigenetic regulation of trained immunity.** Trained immune cells undergo a glycolytic shift that increases pyruvate availability and drives its conversion into acetyl‐CoA and lactate. Lactate promotes histone lactylation (e.g., H3K18la and H3K27la), whereas acetyl‐CoA fuels histone acetyltransferase (HAT) activity to deposit activating acetylation marks such as H3K27ac. Entry of acetyl‐CoA into the tricarboxylic acid (TCA) cycle, together with enhanced glutaminolysis, results in the accumulation of intermediates including succinate, fumarate and malate, which modulate chromatin‐modifying enzymes, notably inhibiting the histone demethylase KDM5. In parallel, increased Set7 and KMT5A activity enhances activating histone methylation (H3K4me and H4K20me1, respectively), while elevated KDM4 combined with reduced EHMT2 promotes removal of repressive marks such as H3K9me3. Reduced sirtuin histone deacetylase (HDAC) activity further preserves activating acetylation states. Together, these coordinated metabolic‐epigenetic changes establish a more accessible chromatin landscape that enhances transcription of pro‐inflammatory genes, thereby sustaining the trained immunity phenotype. Abbreviations: ACLY, ATP citrate lyase; Ac, acetylation; La, lactylation; Me, methylation. Epigenetic enzyme color key: Dark blue, histone methyltransferase; green, histone acetyltransferase; dark pink, histone demethylase; orange, histone deacetylase.

It is well‐established that changes in cellular metabolites act as substrates and cofactors for chromatin‐modifying enzymes, including histone methyltransferases and demethylases, and histone acetyltransferases and deacetylases, leading to the related induction of epigenetic reprogramming at genes involved in innate immune responses (Figure 1).

Epigenetic reprogramming is essential to the establishment and maintenance of long‐term innate immune memory. Indeed, training can be abolished following the inhibition of epigenetic enzymes with methyltioadenosine (MTA, a non‐selective methyltransferase inhibitor) and ITF2357 (ITF, a histone deacetylase inhibitor) [12, 16, 23, 24, 25, 26]. Two of the most well‐studied epigenetic modifications enhanced in trained immunity include histone 3 lysine 4 trimethylation (H3K4me3) at promoters, and histone 3 lysine 27 acetylation at both promoters and distal enhancers of genes involved in immunity/inflammation and metabolism, leading to their increased transcriptional activation in monocytes and macrophages [12, 14, 15, 18, 24, 25, 26, 27, 28, 29, 30, 31]. In line with this, the increase in H3K27ac in β‐glucan‐trained monocytes has been linked to reduced NAD + ‐dependent class III histone deacetylase, Sirtuin‐1, expression [12] Furthermore, the acquisition of H3K4 monomethylation (H3K4me1) at distal enhancers has also been observed in trained immunity [27, 31]. Selective inhibition of H3K4 monomethyltransferase Set7 (SETD7) during training in vitro attenuated pro‐inflammatory cytokine production following restimulation with LPS, and Setd7 knock‐out (KO) mice are unable to mount an enhanced response to secondary challenge in vivo, highlighting the importance of Set7 for trained immunity [28].

In two very recent studies, trained monocytes/macrophages have also been associated with increased histone lactylation. In the first study, transcriptome analysis, ATAC‐seq, and CUT&Tag demonstrated that lactate enhances chromatin accessibility in a manner dependent histone lactylation, specifically lactylation of histone H3 at lysine residue 18 (H3K18la) and 27 (H3K27la), at the promoters of cytokines gene loci, including *IL6* [17]. The second study identified increased H3K18la, mainly at distal regulatory regions, during trained immunity [32]. H3K18la was positively associated with active chromatin and gene transcription, persisted after the elimination of the training stimulus, and was strongly associated with “trained” gene transcription in response to a secondary stimulus [32]. Long‐term histone lactylation persisted in vivo 90 days after vaccination with BCG, and pharmacological inhibition of lactate production or histone lactylation blocked trained immunity responses [32]. However, histone lactylation has previously been demonstrated to act as an endogenous “lactate clock” that directly stimulates gene transcription in the late phase of M1 macrophage polarization to induce the expression of M2‐like homeostatic genes involved in wound healing and repair, including arginase 1 (Arg1) [33]. It is plausible that this mechanism is defunct in trained macrophages that display resistance to induction of anti‐inflammatory processes that mediate regression or repair. Therefore, the role of histone lactylation in trained immunity requires further exploration.

The role of repressive histone modifications in trained immunity has also been explored, for example, repressive histone modification H3K9me3 was found to be decreased at promoters of pro‐inflammatory genes during β‐glucan training, contributing to their increased transcriptional activation [26, 29, 34]. In agreement, the expression of histone methyltransferase G9a, also known as Euchromatic Histone Lysine Methyltransferase 2 (EHMT2), which mediates H3K9 methylation, was decreased following the induction of trained immunity [35]. Pharmacological inhibition of EHMT2 in monocytes amplified trained immunity responses, as shown by increased pro‐inflammatory cytokine production and increased metabolic rate [35]. Moorlag et al. [36] identified a strong association between KDM4 histone demethylases, which promote gene transcription by removing the repressive histone modification H3K9me3, and trained immunity responses. Confirming this, inhibition of KDM4 proteins in vitro by the small molecule JIB‐04 significantly decreased trained immunity responses induced by either β‐glucan or BCG due to increased levels of H3K9me3 at genes important for the induction of glycolysis [36]. Dynamic levels of repressive H3K27me3 were also reported in models of β‐glucan‐induced trained immunity [37]. A small‐molecule catalytic site inhibitor selective for the H3K27me3‐specific demethylase subfamily (KDM6 subfamily members JMJD3 and UTX), called GSK‐J4, reduces LPS‐induced pro‐inflammatory cytokine production by human primary macrophages [38], however, to our knowledge, this has not yet been explored in the context of trained immunity.

Epigenetic mechanisms such as histone modifications are not the only drivers of trained immunity; long noncoding RNAs (lncRNAs) also play a critical role. In particular, immune gene‐priming lncRNAs (IPLs) have emerged as central regulators. Exposure to β‐glucan induces epigenetic reprogramming of immune genes by upregulating IPLs. One example is UMLILO (Upstream Master LncRNA Of The Inflammatory Chemokine Locus), which, when inserted into the chemokine topologically associating domain (TAD) in mouse macrophages, enhances the training of chemokine genes [39]. Within the same TAD, the *IL1B* gene (encoding the pro‐inflammatory cytokine IL‐1β) is positioned alongside *IL37*, which encodes the anti‐inflammatory cytokine IL‐37 that counterbalances IL‐1β activity [40] and has previously been shown to counteract the protective effects of trained immunity in vivo [41]. Recent work has identified an additional lncRNA in this region, AMANZI, which attenuates IL‐1β expression and trained immunity by promoting IL‐37 transcription [40]. Importantly, a common genetic variant, rs16944, within AMANZI strengthens this regulatory axis, thereby predisposing individuals to either heightened pro‐inflammatory responses or immunosuppressive states, depending on context [40].

### 2.3 Peripheral Versus Central Trained Immunity

Initially, trained immunity was discovered in circulating monocytes (i.e., peripheral trained immunity), but the long‐term persistence of trained immunity is reliant upon epigenetic reprogramming at the level of bone marrow progenitor cells that replenish short‐lived peripheral cells (i.e., central trained immunity) [42]. Central trained immunity explains how terminally differentiated cells with short‐half lives, such as macrophages, can also demonstrate “memory” [42, 43]. The longevity of training enables innate immune cells to provide broad and robust protection against a wide range of stimuli but is maladaptive when activated in the context of chronic inflammatory diseases, such as cardiovascular diseases.

## 3 Trained Immunity in Cardiovascular Disease

Cardinal features of trained immunity include long‐term heightened inflammation with increased propensity to generate IL‐1β and IL‐6, which can aggravate ASCVD. IL‐6 will also drive CRP production by the liver. Indeed, trained immunity has been described in ASCVD. For example, Bekkering et al. reported that circulating monocytes isolated from patients with symptomatic atherosclerosis have an enhanced pro‐inflammatory phenotype upon ex vivo LPS stimulation with increased expression of glycolytic enzymes and epigenetic remodeling at the level of histone methylation, specifically decreased H3K27me3 at the TNF‐α promoter compared to control cells [44]. This trained state in peripheral monocytes was accompanied by significantly increased plasma hsCRP compared to controls [44]. Notably, in another study, Zhang et al. demonstrated that cellular markers of trained immunity were increased in monocytes from patients with elevated hsCRP (vs. normal hsCRP) in the context of unstable angina [9]. Specifically, upon ex vivo LPS challenge, monocytes from these patients exhibited heightened pro‐inflammatory cytokine production, metabolic reprogramming (increased glycolysis), and transcriptional and epigenetic reprogramming of proinflammatory genes including NLRP3 inflammasome‐related genes [9]. The findings in this study provide an intriguing association between trained immunity and the clinically useful, but non‐specific, feature of elevated hsCRP and highlight the potential importance of hsCRP as a quantitative integrated marker of trained immunity. That said, trained immunity is a “priming” phenomenon, and its effects are most clearly apparent in the context of an acute stimulus, which was intentionally avoided in these patients with unstable angina, arguing for further in‐depth characterization and prospective monitoring.

Trained immunity has also been reported in patients with AMI without traditional risk factors [8]. The authors found that even in the absence of systemic inflammation (as measured by CRP), circulating monocytes from patients with myocardial infarction (MI) had an increased overall cytokine production capacity following ex vivo LPS stimulation, with the strongest difference reported for IL‐10, which was associated with an enrichment of H3K4me3 at the promoter region [8]. However, RNA sequencing of circulating monocytes revealed no genome‐wide differences in gene expression between patients and controls, suggesting that transcriptional alterations may only become apparent upon restimulation. Future studies examining monocyte responses to secondary challenges, for example, following AMI may provide new insights into the presence and mechanisms of trained immunity. Causally, a recent study also provides evidence that MI itself induces trained immunity in the bone marrow, which exacerbates systemic inflammation and accelerates atherosclerosis development in naïve recipient mice [45]. Mechanistically, blood monocytes and bone marrow–derived macrophages from MI mice showed elevated spleen tyrosine kinase (SYK) expression—a key regulator of pro‐inflammatory signaling through immune receptors (e.g., Dectin‐1)—driven by KMT5A‐mediated H4K20me1 deposition and CCHC‐type zinc finger nucleic acid‐binding protein (CNBP) transactivation [45]. Inhibition of KMT5A or CNBP potentially slowed post‐MI atherosclerosis in vivo. Human relevance was confirmed as classical monocytes from ST‐elevation MI (STEMI) patients with advanced coronary lesions expressed higher SYK and KMT5A gene levels [45]. Indeed, the central roles of SYK and KMT5A in trained immunity have previously been highlighted: heme‐induced training induces SYK phosphorylation in human monocytes [46], while elevated KMT5A expression was observed in blood monocytes from unstable angina patients with hsCRP ≥ 3 mg/L and evidence of trained immunity [9]. Altogether, the association of features of trained immunity with MI plausibly relates AMI with accelerated disease progression/impaired healing, but demonstrating causation and assessing the potential contribution in human observational studies remains challenging, particularly in the absence of robust quantitative clinical biomarkers.

Beyond cytokine and metabolic reprogramming, trained immunity has also been shown to drive maladaptive procoagulant activity; for example, Rehill et al. recently demonstrated that trained immunity also confers a procoagulant phenotype in myeloid cells, mediated by acid sphingomyelinase–dependent tissue factor decryption, thereby linking innate immune memory to immuno‐thrombosis and providing a plausible mechanism through which trained immunity could exacerbate atherothrombotic complications [47].

Taken together, these studies highlight the presence of trained immunity and its association with differences in inflammation risk between individuals—a factor which has been shown to be an independent predictor of cardiovascular events and recurrence.

## 4 Cardiovascular Risk Factors and Trained Immunity

**FIGURE 2:**
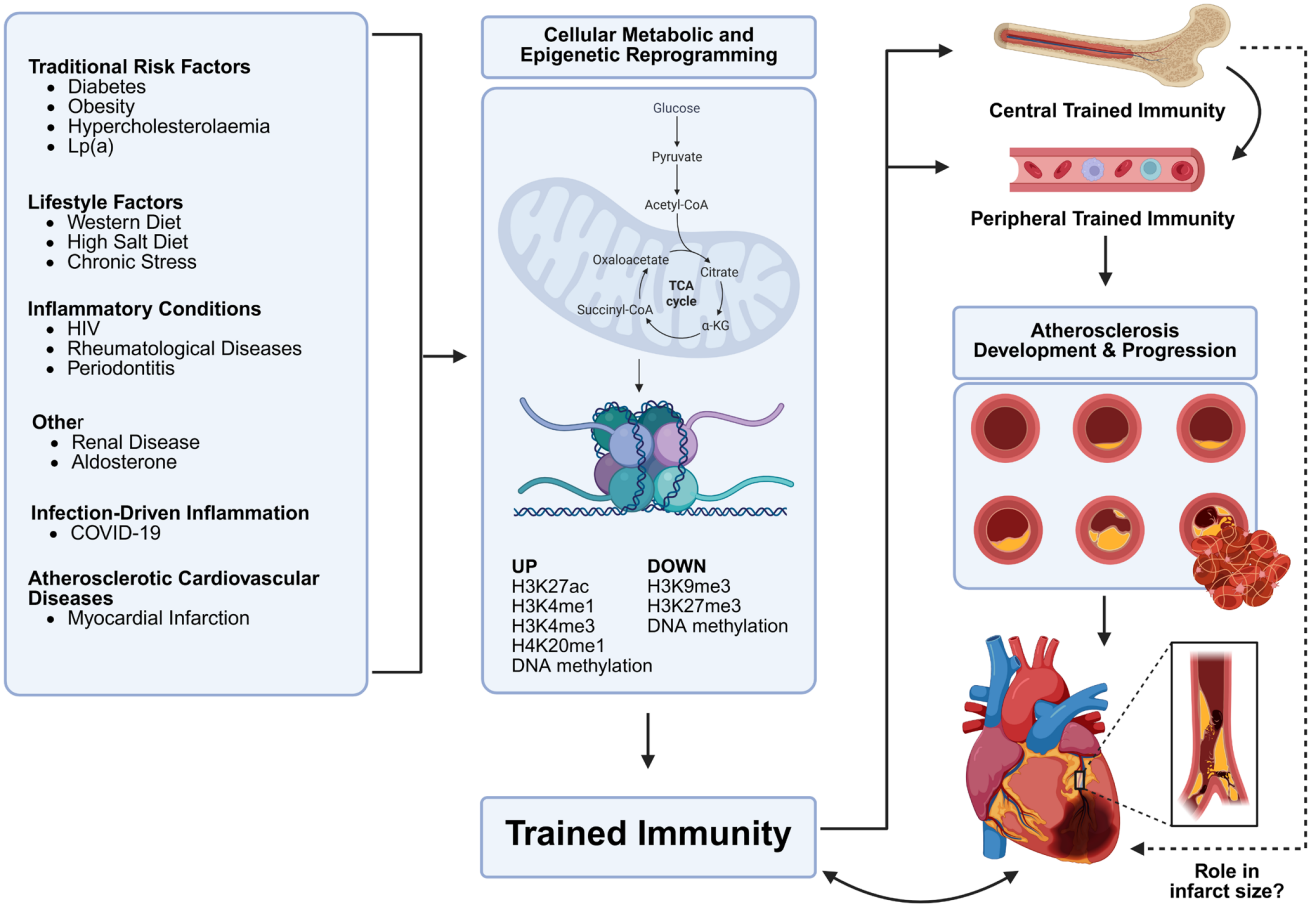
**Drivers and consequences of trained immunity in atherosclerosis** **.** Diverse clinical and environmental stimuli, including traditional cardiovascular risk factors, lifestyle influences, chronic inflammatory conditions, infection‐driven inflammation, and atherosclerotic cardiovascular diseases, contribute to the induction of trained immunity. These factors initiate cellular metabolic and epigenetic reprogramming, involving alterations in key metabolites of the tricarboxylic acid (TCA) cycle and shifts in histone modifications and DNA methylation. The resulting trained immunity phenotype emerges both centrally in bone marrow progenitor cells and peripherally in circulating immune cells. These reprogrammed cells persist over time and drive accelerated atherosclerosis development and progression, ultimately promoting plaque rupture and cardiovascular events, such as myocardial infarction, which itself induces trained immunity, thus perpetuating a vicious cycle of chronic inflammation. Whether the magnitude of central trained immunity influences infarct size remains unknown. Cardiovascular risk factors rarely occur in isolation, raising the possibility that trained immunity induced by multiple factors could be cumulative and build progressively over time.

As well as the presence of trained immunity in cardiovascular disease states, several known risk factors for ASCVD, including diabetes, obesity, hypercholesterolaemia, life‐style factors (e.g., diet and stress), infection, and inflammatory diseases, promote states of trained immunity (summarized in Figure 2).

### 4.1 Diabetes

Diabetes drives vascular complications such as ASCVD and AMI [48], and clinical outcomes for patients who have sustained MI are persistently worse in patients with diabetes [49]. Treatments to prevent the vascular complications of diabetes have largely focused on lowering blood glucose [50], however, this has consistently little or no effect, or markedly delayed effects, in reducing rates of MI [50, 51, 52, 53, 54, 55, 56]. Intriguingly, the UK Prospective Diabetes Study has shown that a period of sub‐optimal glucose control shortly after diagnosis manifests in adverse cardiovascular outcome > 24 years later [57].

This persistent risk of cardiovascular complications in diabetes after glucose‐lowering therapy has been termed ‘metabolic memory’ and may be linked to the development of long‐term hyperglycaemia‐induced trained immunity or ‘HITI’. Of particular interest are observations that hyperglycaemia alters bone marrow function and enhances inflammatory myelopoiesis [58, 59, 60, 61]. For example, diabetic mice have increased numbers of circulating neutrophils and Ly6C^hi^ monocytes, reflecting a hyperglycaemia‐induced proliferation and expansion of bone marrow myeloid progenitors with associated release of monocytes into the circulation [60]. Interestingly, transient intermittent hyperglycaemia (TIH), rather than sustained elevation, promoted myelopoiesis more potently, driving persistent recruitment of inflammatory monocytes into atherosclerotic lesions and accelerating pre‐clinical atherosclerosis [62]. Systemic hyperglycaemia enhanced GLUT1‐dependent glucose uptake in neutrophils, increasing S100A8/S100A9 production, which stimulated myelopoiesis via receptor for advanced glycation end products (RAGE) on common myeloid progenitors. Myeloid‐restricted *Slc2a1* (GLUT1) deletion or pharmacological S100A8/S100A9 inhibition attenuated TIH‐induced myelopoiesis and atherosclerosis. Indeed, plasma S100A8/S100A9 correlates with leukocyte counts and coronary artery disease (CAD) in type 1 diabetes (T1D) [60], suggesting that targeting of the S100A8/S100A9‐RAGE axis may reduce atherosclerosis and cardiovascular events in diabetic individuals with adequate glycaemic control.

In a study of human peripheral blood immune cell profiles from long‐standing T1D patients, as compared to healthy controls, it showed a more active immune profile as demonstrated by a higher percentage and absolute number of neutrophils, monocytes, total B cells, and activated CD4^+^CD25^+^ T cells, while the abundance of regulatory T cells (Tregs) was reduced [63]. Remarkably, a subgroup of T1D patients with diabetes‐related macrovascular complications revealed a stronger proinflammatory phenotype characterized by a lower percentage of FOXP3^+^ Treg, higher proportions of CCR4 expressing CD4 and CD8 T cell subsets, classical, CD196^+^, and CD194^+^ monocyte subsets, a lower Treg/conventional T‐cell ratio, an increased proinflammatory cytokine (TNF‐α, IFN‐γ), and a decreased anti‐inflammatory (IL‐10) producing potential compared to T1D patients without macrovascular complications [63]. Further to this, in CD34^+^ hematopoietic stem and progenitor cells (HSPCs) from sternal bone marrow of CAD patients undergoing bypass surgery, diabetes was associated with upregulation of inflammatory cytokines/chemokines and downregulation of host defense and lymphocyte activation genes [64]. Building on these findings, a subsequent study by the same group revealed that sternal bone marrow‐derived HSPCs from T2DM patients exhibited a senescence‐associated secretory phenotype (SASP) and *SETD7* upregulation, and upon myeloid differentiation, gave rise to senescent proinflammatory intermediate monocytes (CD14^++^CD16^+^), characterized by H3K4me1 enrichment at the Nuclear factor kappa‐light‐chain‐enhancer of activated B cells (NF‐κB)‐p65 promoter [65].

Mechanistically, hyperglycaemia has been shown to alter the function of bone marrow cells and their macrophage progeny through mechanisms that depend on both metabolic and epigenetic reprogramming. For example, high extracellular glucose results in a shift towards glycolysis and accumulation of TCA cycle intermediates with consequent enhanced pro‐inflammatory and pro‐atherogenic gene expression and decreased anti‐inflammatory gene expression in mouse macrophages [13]. Crucially, these changes persist even after the cells are subsequently restored to a normal physiological glucose environment. Inhibition of glycolysis, using dichloroacetate and 2‐DG, inhibited changes in inflammatory gene expression and pro‐atherogenic functions [13]. Furthermore, hyperglycaemia reprograms hematopoietic stem cells (HSCs) culminating in distinct chromatin modifications (e.g., histone 3 lysine 4 trimethylation and histone 3 lysine 27 acetylation) in HSCs and their macrophage progeny [6, 13, 66]. This “HITI” promotes atherosclerosis and persists in vivo despite complete correction of hyperglycaemia [13], confirming a cardiovascular disease‐relevant and persistent form of trained immunity. Equivalent findings of “HITI” were confirmed in atherosclerotic plaque macrophages and circulating PBMCs from patients with type 2 diabetes.

Further studies investigate the nature of epigenetic reprogramming in peripheral monocytes exposed to hyperglycaemic conditions. For example, THP‐1 monocytes cultured in conditions of high extracellular glucose (25 mM) led to increases in H3K4me2 and H3K9me2 at promoter regions of genes associated with chronic inflammation, including *IL1A* and *IL8*, which were confirmed in peripheral monocytes isolated from patients with type 1 and type 2 diabetes [67]. Moreover, high glucose culture of monocytes, mimicking diabetic conditions, can recruit the key p65 subunit of NF‐κB and histone acetyltransferases to the promoters of inflammatory genes such as TNF‐α and Cyclooxygenase 2 (COX‐2), resulting in histone acetylation (e.g., H3K9ac, H3K14ac, H4K5ac, H4K8ac, and H4K12ac), chromatin remodeling, and increased transcriptional activation [68]. These changes were confirmed in monocytes isolated from patients with type 1 and type 2 diabetes. Notably, overexpression of histone deacetylase isoforms inhibited the p65‐mediated up‐regulation of TNF‐α transcription [68]. Additional studies of hyperglycaemia‐induced training of monocytes from patients with type 1 diabetes demonstrated that epigenetic reprogramming was dependent upon up‐regulation of the mixed lineage leukemia (MLL) lysine methyltransferases, which methylate H3K4 [23]. Treatment with the MLL inhibitor, menin‐MLL, during the process of trained immunity repressed the pro‐inflammatory phenotype. Other significantly up‐regulated methyltransferases include *SETD1A* and *SETD1B*; however, these were not further explored [23].

Overall, multiple studies demonstrate that high glucose reprogrammes HSPCs and monocytes via metabolic and epigenetic mechanisms, promoting enhanced myelopoiesis, sustained pro‐inflammatory cytokine production, and accelerated atherosclerosis despite restoration of normoglycaemia. These findings highlight an important role for HITI in the perpetuation of chronic inflammation and acceleration of ASCVD progression in patients with diabetes, while simultaneously impairing processes of ASCVD regression or repair. This may help to explain why glucose‐lowering therapy alone, without also targeting processes of inflammation, is ineffective in reducing cardiovascular risk. Nonetheless, the precise extent, duration, and patterns of hyperglycaemia that confer this heightened risk remain to be defined.

### 4.2 Obesity

Obesity is, in part, a chronic inflammatory disorder and is an independent risk factor for cardiovascular disease. In a recent study, adolescents with obesity had increased systemic and cellular inflammation compared to controls, which were associated with worse subsequent subclinical cardiovascular measures [44]. Adolescents with obesity had increased monocyte activation and cytokine production upon stimulation, and monocyte transcriptomics demonstrated upregulated inflammatory pathways and downregulated anti‐viral responses [44]. In another study, accumulation of pro‐inflammatory adipose tissue macrophages (ATMs) in white adipose tissue mass was associated with obesity‐induced inflammation [69]. This deleterious phenotypic switch of innate immune cells persists even after obesity‐associated metabolic alterations have been normalized through weight loss, which has been termed an “obesogenic memory” [70]. Indeed, in patients with obesity, systemic inflammatory markers and leukocyte counts fall to levels comparable with healthy lean individuals within 6 months of bariatric surgery; however, monocytes retain a residual functional and transcriptional hyper‐inflammatory phenotype [71]. This may be driven, at least in part, by the ability of adipose tissue to imprint long‐term pro‐inflammatory memory in monocytes. Further evidence demonstrates that this obesity‐induced trained immunity is exacerbated by complete or partial weight regain (termed weight cycling) as evidenced by elevated metabolism and enhanced cytokine production in adipose tissue macrophages, which further accelerates cardiometabolic disease [72, 73]. Interestingly, it has been demonstrated in vitro that the distribution of adipose tissue in obese patients can differentially induce trained immunity in peripheral monocytes (Abstract: doi.org/10.1093/eurheartj/ehab724.3438). Specifically, both visceral adipose tissue (VAT) and subcutaneous adipose tissue (SAT) from patients with obesity induced persistent innate immune cell activation in healthy human monocytes, as assessed by increased cytokine production in response to a secondary stimulus. However, adipose tissue‐secreted metabolites from VAT induced a higher cytokine response when compared to SAT, suggesting that VAT has an enhanced potential to induce trained immunity. Strategies that focus on the reduction of VAT in obese subjects might help to reduce the severity of obesity‐associated trained immunity and its cardiometabolic consequences.

Sex differences in inflammatory status have also been reported in obese individuals. In obese women with metabolic syndrome, authors identified a lower concentration of the anti‐inflammatory adiponectin, whereas in obese men, the presence of metabolic syndrome is associated with higher circulating leptin and IL‐6 concentrations and increased PBMC cytokine production capacity [74]. In a follow‐up study, leptin was found to significantly positively correlate with circulating IL‐1β and IL‐6 levels, and treatment of healthy human monocytes with leptin induced long‐term monocyte hyperresponsiveness in an in vitro model of trained immunity [75]. Thus, these data warrant further investigation into the role of leptin in the regulation of trained immunity and cardiometabolic disease in obesity.

Mechanistically, obesogenic memory is dependent upon both metabolic and epigenetic reprogramming. For example, the treatment of healthy mouse macrophages with palmitic acid or adipose tissue conditioned media from obese mice significantly increased maximal glycolysis and oxidative phosphorylation and increased LPS‐induced TNF‐α and IL‐6 production in vitro in a manner dependent upon TLR4 signaling [72]. These effects were impaired by inhibition of mTOR (metformin) or methyltransferase inhibition (MTA), thus confirming this form of innate memory is driven by metabolic and epigenetic changes [72]. As with other forms of trained immunity, obesity also reprogrammes myeloid cells at the level of the bone marrow [76], driving both quantitative increases in myeloid progenitors and the preferential generation of inflammatory ATMs even after serial bone marrow transplantation, an effect that was regulated by hematopoietic MyD88 [76]. As in TIH, obesity enhanced myelopoiesis in the bone marrow via adipose‐derived production of S100A8/S100A9 [77]. This induced ATM TLR4/MyD88 and NLRP3 inflammasome‐dependent IL‐1β production, which interacted with the IL‐1 receptor on myeloid progenitor cells to stimulate the enhanced production of monocytes and neutrophils [77]. This highlights a potential positive feedback mechanism in obesity, whereby the inflamed adipose tissue stimulates the production of more pro‐inflammatory monocytes, which perpetuates further inflammation. Thus, targeting of the NLRP3‐IL‐1β signaling axis could reduce adipose tissue inflammation and cardiometabolic disease in obesity.

### 4.3 Hypercholesterolaemia

Patients with hypercholesterolaemia have an increased risk for ASCVD. In untreated patients, the risk is strongly related to the level of LDL‐cholesterol. However, in the context of effective lipid‐lowering with statin therapy, the relationship with LDL‐cholesterol is largely lost, but important residual risk is strongly associated with the level of hsCRP, as an indicator of inflammation [3].

Bekkering et al. [78] found that monocytes from patients with familial hypercholesterolaemia are characterized by a trained immune phenotype. Importantly, lowering cholesterol levels using a 3‐month statin treatment did not reverse monocyte hyperresponsiveness in this patient population, with key epigenetic marks including increased H3K4me3 and decreased H3K9me3 at the promoters of pro‐inflammatory cytokines (e.g., TNF‐α) remaining unaffected by statin treatment [78]. As with hyperglycaemia, the effects of hypercholesterolaemia‐induced reprogramming were detectable at the level of the bone marrow progenitor cells. For example, bone marrow aspirates from patients with familial hypercholesterolaemia taken before and 3 months after cholesterol‐lowering statin therapy showed increased gene expression in pathways involved in hematopoietic migration and myelomonocytic skewing [79]. Interestingly, statin therapy reversed myelomonocytic skewing, but the transcriptomic reprogramming of monocyte‐associated inflammatory and migratory pathways persisted, indicating the presence of central trained immunity [79].

Further verification of the effects of hypercholesterolaemia on bone marrow progenitor cells and its clinical relevance to ASCVD has been evidenced in pre‐clinical models. For example, the activation of hematopoietic progenitor cell proliferation and skewed development towards myeloid lineages—especially granulocytes and inflammatory monocytes—persisted following bone marrow transplantation from hypercholesterolaemic Ldlr^−/−^ mice into normocholesterolaemic recipients, which resulted in increased leukocyte migration into the artery, with consequent significantly increased plaque size with more advanced characteristics [80]. These data confirm the cardiovascular disease‐relevant nature of hypercholesterolaemia‐induced training and provide a further rational basis targeting inflammation to lower residual cardiovascular risk in patients with hypercholesterolaemia in addition to statin therapy.

Furthermore, atherogenic compound, oxLDL, resulting from the oxidation of LDL cholesterol can directly activate macrophages to induce a prolonged pro‐inflammatory and pro‐atherogenic phenotype which is sustained by metabolic and epigenetic reprogramming in human monocytes, and associated with an upregulation of scavenger receptors CD36 and scavenger receptor‐A and downregulation of ATP‐binding cassette transporters, ABCA1 and ABCG1 [24]. Keating et al. [14] identified that oxLDL‐induced training is critically dependent on intracellular metabolic alterations, including a concomitant upregulation of glycolysis and oxidative phosphorylation as indicated by increased oxygen consumption rate. In support of this, oxLDL‐induced trained immunity was found to induce transcriptional activation of genes enriched in mitochondrial metabolic pathways, and metabolome analysis revealed mitochondrial TCA cycle as the most upregulated pathway [81]. Indeed, oxLDL‐induced trained immunity is associated with changes in mitochondrial size, mass, and membrane polarization [81]. The importance of the mitochondria in supporting cytokine hyperresponsiveness in trained immunity was confirmed using pharmacological inhibitors targeting mitochondrial function, which dose‐dependently inhibited the production of TNF‐α [81]. Notably, decreasing mitochondrial stress in macrophages can prevent inflammation in ASCVD and decrease atherosclerosis burden in mice [82], thus representing a potential therapeutic avenue to treat trained immunity in cardiometabolic disease.

In addition to oxLDL, Lp(a), the major lipoprotein carrier of phosphocholine‐containing oxidized phospholipids (OxPLs) in plasma, has also been associated with the induction of trained immunity in monocytes as evidenced by an increased capacity to transmigrate and produce pro‐inflammatory cytokines upon stimulation [83]. Moreover, in vitro studies found that Lp(a) augments the pro‐inflammatory response in healthy human monocytes, which was markedly attenuated by inactivating OxPL on Lp(a), which is a recognized DAMP. This is clinically relevant as subjects with elevated Lp(a) have increased arterial inflammation and enhanced PBMC trafficking to the arterial wall, suggesting that trained immunity might provide a novel link between Lp(a), OxPL, and accelerated atherosclerosis in humans.

### 4.4 Western Diet

Western‐type calorically rich diets (WD), containing numerous immunologically active substances (e.g., high glucose, cholesterol, saturated fatty acids, L‐carnitine and phosphatidylcholine, which are converted to trimethylamine N‐oxide (TMAO)), are proficient at inducing a chronic inflammatory state that is associated with long‐term innate immune cell reprogramming [84]. Specifically, in atheroscelrotic prone Ldlr^−/−^ mice a 4‐week WD induced the transcriptomic and epigenomic reprogramming of myeloid progenitor cells with skewing towards myelopoiesis, which persisted after 4 subsequent weeks of chow‐diet and resulted in a hyperresponsive phenotype upon restimulation [85]. Mechanistically, the authors identified the NLRP3 inflammasome and subsequent production of IL‐1β as criticial to the WD‐induced trained immunity phenotype. *Nlrp3* deletion in Ldlr^−/−^ mice led to the abolition of WD‐induced systemic inflammation, hematopoiesis and myeloid precursor reprogramming. Thus, NLRP3 orchestrates WD‐induced training and could be targeted to mitigate the deleterious complications of the WD such as cardiovascular disease. The relevance of WD‐induced trained immunity to the development of ASCVD is demonstrated by the significantly increased aortic root plaque size (in the absence of changes in serum cholesterol) following bone marrow transplantation from WD‐fed Ldlr^−/−^ mice into chow‐fed recipients [86]. Mice reconstituted with WD‐fed bone marrow exhibited hypomethylation of CpG regions in the genes encoding *Pu.1* and interferon regulatory factor 8 (*Irf8*), key transcriptional regulators of monocyte proliferation and macrophage differentiation, and increased numbers of circulating peripheral leukocytes [86].

### 4.5 High‐Salt Diet

Excess dietary salt is a well‐established risk factor for hypertension, which in turn accelerates ASCVD and heart failure [87]. Beyond its hemodynamic effects, a high‐salt diet has recently been identified as a novel inducer of trained immunity [88]. In experimental models, a high‐salt diet induces innate immune priming and training in HSPCs, leading to enhanced inflammatory responses but impaired reparative capacity. Mechanistically, this training involves downregulation of the *NR4a* transcription factor family, particularly *NR4a1*, alongside impaired mitochondrial oxidative phosphorylation [88]. Healthy mice transplanted with bone marrow from high‐salt diet‐fed mice retain signatures of reduced reparative functions, further confirming a persistent form of innate immune memory that originates in the bone marrow. Functionally, in models of intracerebral hemorrhage, high‐salt diet‐induced training impairs reparative macrophage polarization, resulting in defective hematoma clearance and worsened stroke recovery [88]. In parallel, sodium exposure has been shown to exacerbate mTORC1‐dependent trained immunity in mature macrophages, reinforcing a hyper‐responsive, pro‐inflammatory phenotype that accelerates chronic kidney disease progression [89]. Thus, a high‐salt diet can both initiate and potentiate ongoing training in myeloid cells during chronic inflammatory conditions, highlighting why excess salt is such a potent and persistent risk factor for cardiovascular and cerebrovascular disease.

### 4.6 Chronic Stress

Exposure to physchological stress is associated with systemic inflammation [90] and is a risk factor for atherosclerosis [91], MI [92], and death from MI [93]. Of particular interest are observations that chronic stress alters bone marrow function and enhances inflammatory myelopoiesis. For example, Heidt et al. demonstrated that chronic stress leads to enhanced proliferation of HSPCs and consequent increased output of disease‐promoting neutrophils and inflammatory monocytes in humans [94]. Mechanistically, the authors demonstrated that during chronic stress in mice, sympathetic nerve fibers release surplus noradrenaline, which signaled bone marrow niche cells to decrease levels of HSC retaining factor CXCL12 through the β_3_‐adrenergic receptor [94]. Atherosclerosis‐prone Apoe^−/−^ mice subjected to chronic stress showed accelerated hematopoiesis with a myeloid bias, which accelerated atherosclerosis and promoted plaque features associated with vulnerable lesions that cause MI and stroke in humans [94]. Also, it should be noted that MI itself results in sympathetic nervous system activation and increase in (nor)adrenaline levels, therefore, this might represent another mechanism by which MI drives processes of inflammation and accelerates atherosclerosis [95].

Furthermore, the effects of catecholamines (adrenaline and noradrenaline) on myeloid progenitors has been linked to the induction of trained immunity [96]. The authors identified that monocytes transiently exposed to adrenaline and noradrenaline undergo metabolic reprogramming (increased glycolysis) and mount a persistent hyperresponsive proinflammatory phenotype that is again dependent upon the β‐adrenergic receptor [96]. This catecholamine‐induced trained immunity phenotype was confirmed in pheochromocytoma patients, who have paroxysmal catecholamine excess, and was hypothesized to contribute to the increased cardiovascular risk in this patient population [96]. As well as the β‐adrenergic receptor, increased myelopoiesis and chromatin and transcriptional reprogramming of bone marrow monocytes following psychological stress in mice has been linked to the Akt–mTOR‐HIF‐1α and interferon (IFN) signaling pathways, which are known to be critical mediators of the metabolic and epigenetic rewiring of myeloid cells, respectively [97]. Potential clinical relevance was supported by a trained phenotype in monocytes isolated from human subjects with high vs. low stress, which may explain how psychological stress confers inflammatory disease risk.

### 4.7 Chronic Inflammatory Diseases

#### 4.7.1 HIV

ASCVD and its most fatal complication, MI, is a leading cause of morbidity and mortality among people living with HIV [98]. Across the different cardiovascular disease manifestations, a common pathogenic feature is HIV‐associated inflammation, including elevated biomarkers of systemic inflammation (e.g., CRP and IL‐6) and monocyte activation [99, 100]. Specifically, in a study of 211 people living with HIV on stable antiretroviral therapy, monocytes exhibited exacerbated cytokine responses to ex vivo restimulation, particularly of IL‐1β, compared with HIV‐uninfected controls, and this response correlated with plasma hsCRP levels [101]. Transcriptomics confirmed priming of the monocyte IL‐1β pathway, consistent with a monocyte‐trained immunity phenotype. Notably, the increased monocyte responsiveness persisted for more than 1 year [101]. In an in vitro model of HIV trained immunity, human monocyte‐derived macrophages originating from healthy monocytes treated with extracellular vesicles containing HIV‐1 protein Nef (exNef) for 48 h, but differentiated in the absence of exNef, released increased levels of pro‐inflammatory cytokines after LPS restimulation [102]. The authors observed chromatin changes at genes involved in inflammation and cholesterol metabolism pathways. Bone‐marrow‐derived macrophages from exNef‐injected mice, as well as from mice transplanted with bone marrow from exNef‐injected animals, produced elevated levels of TNF‐α upon stimulation [102]. Therefore, trained immunity might provide a possible explanation for chronic inflammation in some HIV‐infected individuals treated with anti‐retroviral therapy, and provides a promising therapeutic target for inflammation‐related comorbidities.

#### 4.7.2 Rheumatological Diseases

The chronic rheumatic autoimmune disease primary Sjögren's syndrome (pSS) is associated with accelerated (subclinical) atherosclerosis and other cardiovascular risk factors [103, 104], which is also seen in other autoimmune rheumatic diseases, including systemic lupus erythematosus (SLE) [105], and rheumatoid arthritis [106]. Notably, a phenoptype indicative of trained immunity was identified in PBMCs from patients with pSS [107]. Specifically, PBMCs from pSS patients had increased glucose consumption compared with controls, and produced more TNF‐α upon LPS stimulation, however this did not reach significance [107]. In a study of SLE, Yanginlar et al. demonstrated that plasma isolated from SLE patients can induce trained immunity in monocytes in vitro, as determined by increased IL‐6 production following restimulation [108]. Furthermore, PBMCs from SLE patients in remission produced more IL‐6, IL‐1β, and TNF‐α upon stimulation, accompanied by increased monocyte expression of inflammatory/metabolic genes and altered H3K4me3 [108]. Although not explicitly described as trained immunity, patients with rheumatoid arthritis display increased frequencies of CD14^+^ monocytes that exhibit a hyper‐inflammatory and hyper‐metabolic phenotype upon ex vivo LPS restimulation compared with healthy controls [109]. Inhibition of glycolysis with 2‐DG markedly reduced the expression of pro‐inflammatory cytokines and chemokines, including TNF‐α, IL‐6, IL‐1β, CXCL10, and CXCL11. Mechanistically, the study identified signal transducer and activator of transcription 3 (STAT3) as a central mediator of this phenotype, as selective STAT3 inhibition significantly attenuated both inflammatory and metabolic responses [109]. These findings suggest a persistent reprogramming of innate immunity in rheumatoid arthritis, and an in‐depth analysis of transcriptomic and epigenomic profiles would be valuable to confirm whether this reflects a bona fide trained immunity state.

Moreover, hyperuricemia is a metabolic condition intrinsic to gout pathogenesis, one of the most common forms of inflammatory arthritis, which is associated with increased risk of cardiovascular diseases [110]. Monocytes from healthy volunteers were primed with uric acid and then subjected to stimulation with LPS in the presence or absence of monosodium urate crystals, this led to significantly enhanced gene expression and secretion of IL‐1β and suppression of antagonist interleukin‐1 receptor antagonist (IL‐1Ra) [111]. Mechanistically, uric acid activated the Akt–PRAS40 pathway and mTOR, which inhibits autophagy and activates inflammation [111].

#### 4.7.3 Periodontitis

There is an enhanced risk of systemic diseases (e.g., cardiometabolic disease and arthritis) in periodontitis [112], which is an inflammatory disease of the soft and bone tissues that support the dentition that causes chronic low‐grade systemic inflammation. Experimental‐periodontitis‐related systemic inflammation in mice induced epigenetic reprogramming of HSPCs and led to elevated production of myeloid cells with an increased inflammatory phentoype [113]. Furthermore, the periodontitis‐induced trained phenotype was transmissible by bone marrow transplantation to naive recipients, which exhibited increased inflammatory responsiveness and disease severity when subjected to inflammatory arthritis [113]. The authors identified IL‐1 signaling in HSPC as essential for maladaptive training by periodontitis. Periodontitis is driven by a dysbiotic oral microbiome in which *P. gingivalis* is a major contributor. Notably, *P. gingivalis* has been shown to directly induce trained immunity in human PBMCs in vitro, evidenced by an increased capacity to produce IL‐6 and TNF‐α [114]. However, in this study, whilst circulating IL‐6 and IL‐1Ra concentrations were generally higher in patients with severe periodontitis, ex vivo restimulation of PBMCs did not show a hyperresponsive phenotype and no differences were observed for vascular inflammation as visualized by PET/CT scan compared to controls [114]. Therefore, further investigation into maladaptive innate immune training in periodontitis and its inflammatory comorbidities is required.

### 4.8 Other

#### 4.8.1 Renal Disease

Cardiovascular disease is a leading cause of death among patients with end‐stage renal disease [115], and uremic toxins have been associated with cardiovascular risk and mortality due to their ability to generate oxidative stress and a proinflammatory cytokine milieu [116]. A major uremic toxin, indoxyl sulfate, has been associated with the development of CKD‐related complications such as cardiovascular disease [117]. Indoxyl sulfate has recently been reported to induce trained immunity in monocytes via epigenetic and metabolic reprogramming, resulting in an augmented cytokine production, which was completely abolished by inhibition of glycolysis and methyltransferase activity [16]. Notably, ex vivo monocytes of end‐stage renal disease patients had a higher production of pro‐inflammatory cytokines following LPS stimulation [16]. Mechanistically, the aryl hydrocarbon receptor contributes to indoxyl sulfate‐trained immunity by enhancing the expression of arachidonic acid metabolism‐related genes such as arachidonate 5‐lipoxygenase (ALOX5) and ALOX5 activating protein (ALOX5AP) [16].

In patients with diabetic kidney disease, Chen et al. reported that PBMCs exhibit increased expression of DNA methyltransferase 1 (DNMT1), leading to aberrant hypermethylation of promoter regions of genes that regulate the mTOR pathway [118]. This suppressed negative regulators of mTOR, thereby heightening mTOR signaling and inflammatory activation, hallmarks of trained immunity [118]. Inhibition or knockdown of DNMT1 restored methylation patterns, dampened mTOR activity, and reduced inflammatory gene expression [118]. More recently, a study of kidney transplant recipients found that serum collected 1 week after transplantation can suppress trained immunity, and recipients whose serum exhibited the strongest suppressive capacity rarely experienced graft loss [119]. The authors hypothesized that this effect was mediated by previously unreported effects of immunosuppressive drugs [119]. These findings suggest that, immunosuppressive drugs, beyond improving kidney allograft survival, might also confer cardiovascular benefits via targeting of trained immunity. However, despite kidney transplantation reducing the overall cardiovascular burden of ESRD, cardiovascular disease still remains the leading cause of premature mortality and allograft loss, suggesting a more complex mechanism exists beyond trained immunity [120].

#### 4.8.2 Hormones

In a different but related context, supranormal levels of hormone aldosterone (hyperaldosteronism) are associated with an increased cardiovascular risk in humans [121], and with accelerated atherosclerosis in animal models [122]. Hyperaldosteronism can be caused by autonomous adrenal overproduction of aldosterone [primary hyperaldosteronism (PHA)], one of the most prevalent forms of secondary hypertension [123], or result from general activation of the renin‐angiotensin‐aldosterone system (RAAS), as commonly seen in renal and cardiac failure and obesity. Importantly, clinical data from patients with PHA revealed that chronic exposure to supranormal levels of aldosterone is associated with an increase in cardiovascular events independent of the presence of hypertension [121]. It has been demonstrated that aldosterone augments proinflammatory cytokine production and reactive oxygen species production in monocyte‐derived macrophages after restimulation, via the mineralocorticoid receptor [124]. These effects were not mediated by either induction of glycolysis or oxidative phosphorylation but was epigenetically mediated via enrichment of H3K4me3 at promoters of genes central to the fatty acid synthesis pathway, and pharmacological inhibition of this pathway blunted aldosterone‐induced trained immunity [124]. Thus, trained immunity may provide a novel mechanistic link between aldosterone and cardiovascular disease.

### 4.9 Infection‐Driven Inflammation

#### 4.9.1 Covid‐19

One intriguing aspect of COVID‐19, the severe respiratory illness associated with SARS‐CoV‐2 infection, is the markedly increased rate of cardiovascular disease complications and post‐acute risk of thrombotic events observed in patients, such as MI and stroke [125, 126]. Intriguingly, circulating leukocytes from the blood of COVID‐19 patients showed increased responses to TLR ligands as measured by enhanced cytokine release, namely IL‐1β, IL‐6, and TNF‐α, suggestive of trained immunity [127]. Additionally, it has been demonstrated that inactivated SARS‐CoV‐2 can induce properties associated with trained immunity in human monocytes [128]. Further to this, SARS‐CoV‐2 spike protein (S‐protein) was found to prime inflammasome formation and release of mature IL‐1β in macrophages derived from COVID‐19 patients but not in macrophages from healthy SARS‐CoV‐2 naïve individuals, which correlated with distinct epigenetic and gene expression signatures suggesting innate immune memory after recovery from COVID‐19 [129].

## 5 Immunomodulatory Trials in Cardiovascular Disease

Several recent randomized, controlled trials have shown a reduction in cardiovascular events with immunomodulating drugs (Table 1). Significantly, the use of anti‐inflammatory therapeutics targeted against IL‐1β (The Canakinumab Anti‐Inflammatory Thrombosis Outcome Study [CANTOS]) lead to a reduction in cardiovascular events independently of lipid lowering in patients with previous MI and elevated hsCRP [130]. Furthermore, subsequent analyses showed that the magnitude of hsCRP reduction [131] or IL‐6 reduction [132] below a threshold was directly related to a reduction in major adverse cardiovascular events. Therefore, monitoring of hsCRP or IL‐6 might provide a simple clinical method to identify individuals most likely to accrue the largest benefit from continued immunomodulatory treatment. Moreover, RESCUE, a phase 2 trial, showed marked reductions in hsCRP in response to the anti‐IL‐6 ligand antibody, ziltivekimab, among patients with high cardiovascular risk [133]. Based on these data, an ongoing phase 3 trial, ZEUS (recruitment completed), investigates the effect of ziltivekimab in patients with chronic kidney disease, increased hsCRP, and established cardiovascular disease (https://clinicaltrials.gov/study/NCT05021835). In addition, another ongoing phase 3 trial, ARTEMIS (recruitment ongoing), investigates the effects of ziltivekimab in patients with AMI (https://www.clinicaltrials.gov/study/NCT06118281).

**TABLE 1:** Summary of clinical trials investigating immunomodulatory therapies in cardiovascular disease.

Clinical trial	Year	Phase	Drug	Target	Population	Clinical outcome	References
CANTOS	2019	3	Canakinumab	IL‐1β	Previous MI and elevated hsCRP	Reduced cardiovascular events, independent of lipid‐level lowering	[130, 131, 132]
RESCUE	2020	2	Ziltivekimab	IL‐6	High CV risk	Reduced biomarkers of inflammation and thrombosis relevant to atherosclerosis	[133]
ZEUS	Active	3	Ziltivekimab	IL‐6	CKD, increasing hsCRP and established CVD	Not completed	https://clinicaltrials.gov/study/NCT05021835
ARTEMIS	Active	3	Ziltivekimab	IL‐6	Patients with AMI	Not completed	https://www.clinicaltrials.gov/study/NCT06118281
OXI pilot trial	2019	4	Hydroxychloroquine	Non‐specific	Patients with MI	Reduced IL‐6 levels	[134]
CIRT	2019	3	Low‐dose MTX	Non‐specific	CAD	No reduction in IL‐1β, IL‐6, or CRP and did not result in fewer cardiovascular events	[135]
COLCOT	2019	3	Colchicine	Non‐specific	MI	Reduced ischemic cardiovascular events	[136]
LoDoCo2	2020	3	Colchicine	Non‐specific	Chronic coronary disease	Reduced cardiovascular events	[137]
OASIS‐9/CLEAR SYNERGY	2024	3	Colchicine	Non‐specific	MI with PCI	No reduction in death from cardiovascular causes, recurrent myocardial infarction, stroke, or unplanned ischemia‐driven coronary revascularization	[138]
IVORY	2023	2	Aldesleukin	IL‐2	ACS and hsCRP > 2 mg/mL	Increased circulating Tregs	[139, 140]
IVORY‐FINALE	Active	Observational	Aldesleukin	IL‐2	Participants of IVORY Trial	Not completed	https://clinicaltrials.gov/study/NCT06427694

Interestingly, a randomized, controlled pilot trial of hydroxychloroquine (OXI pilot trial) in patients after MI significantly reduced IL‐6 levels [134]. Hydroxychloroquine use in rheumatology patients has been associated with reductions in cardiovascular event rates [141, 142, 143], but adverse effects have been reported in patients with pre‐existing heart failure [144]. Moreover, the CIRT (Cardiovascular Inflammation Reduction Trial) trial, low‐dose methotrexate (a non‐specific anti‐inflammatory agent) failed to reduce recurrent vascular events in patients with coronary disease [135]. In that trial, no reduction of IL‐1β, IL‐6, or CRP was observed, suggesting that future trials should more specifically target pathways that are more tightly linked to atherosclerosis [135].

Colchicine binds to and inhibits tubulin polymerization and microtubule formation, resulting in multiple anti‐inflammatory effects, including inhibition of NLRP3 inflammasome activity. In patients with recent MI (COLCOT) [136] or chronic coronary disease (LoDoCo2) [137], who were not selected according to evidence of inflammation, colchicine treatment led to a significantly lower rate of major adverse cardiovascular events. However, the OASIS‐9/CLEAR SYNERGY clinical trial in patients following percutaneous coronary intervention (PCI) for ST‐segment elevation or large non–ST‐segment elevation MI showed no difference for colchicine vs. placebo [138]. Clinical uptake has not been universal and the experience with colchicine also argues for deeper mechanistic insight and more precise selection to match patient/target with drug action.

A recent approach has explored the potential to modulate pathogenic processes of inflammation by harnessing the activity of immunomodulatory Treg population [145]. A small (*n* = 60) double‐blind, randomized, placebo‐controlled, phase II clinical trial, IVORY, assessed the effects of low‐dose IL‐2 (Aldesleukin) on vascular inflammation in patients with ACS with hsCRP levels ≧ 2 mg/L [139]. Low‐dose IL‐2 significantly increased circulating Tregs compared to placebo, without causing T effector activation [140]. Vascular inflammation appeared reduced on [145] FDG‐positron emission tomography (PET). Larger trials are needed to confirm its impact on cardiovascular outcomes (IVORY‐FINALE; https://clinicaltrials.gov/study/NCT06427694).

## 6 Conclusions and Future Perspectives

Systemic inflammation is increasingly recognized as central to the pathogenesis of ASCVD and its complications. Treatments of inflammation have reported benefit in the context of established ASCVD (e.g., IL‐1β) and further clinical trials are ongoing. However, enthusiasm for broadly using imprecisely targeted “anti‐inflammatory” drugs is limited by concerns about infection risk and the high cost of expensive biologics in populations not identified as likely to respond, highlighting the need for more targeted immunomodulation as part of the next‐generation therapies.

Thus, elucidating the underlying sources of systemic inflammation—here proposed to include trained immunity—will yield new opportunities for targeted treatment and prevention. Notably, ASCVD risk factors rarely occur in isolation, raising the possibility that trained immunity induced by multiple factors could be cumulative and build progressively over time. On the other hand, processes of trained immunity might be disproportionate in patients with ASCVD and a paucity of conventional risk factors.

Support for these concepts comes from studies of epigenetic clocks, which show that DNA methylation changes accumulate throughout life in response to diverse aging‐related insults and serve as robust markers of biological age [146]. By analogy, cumulative epigenetic reprogramming driven by cardiovascular risk factors may underlie and amplify persistent alterations in innate immune function, thereby sustaining chronic inflammation and accelerating ASCVD progression.

In this review, we highlighted the mechanisms that could associate trained immunity to the development and progression of ASCVD. Deeper insights into metabolic and epigenetic mechanisms driving the long‐lasting effects of trained immunity could enable the development of innovative targeted therapies for ASCVD. Although CRP remains a widely used clinical correlate of systemic inflammation, it is a broad and non‐specific marker and cannot alone characterize trained immunity. Future work should aim to delineate shared mechanisms and potential cumulative effects of trained immunity, through leveraging large ‘‐omics’ datasets, shared repositories, and advanced analytical tools, to inform more precise strategies for targeting processes of inflammation in ASCVD prevention and treatment.

## Conflicts of Interest

R.P.C. is the UK Chief Investigator for the ZEUS trial (sponsor Novo Nordisk) and serves on the Global Expert Panel for that trial. He is engaged by Oxford University Consulting to undertake paid consultancy work for Novo Nordisk, NodThera, Velakor, Tourmaline Bio, and Ventyx Biosciences. He serves on the Scientific Advisory Board of Tourmaline Bio. His laboratory receives, or has received, research funding from the Novo Nordisk Foundation, Novo Nordisk Research Centre Oxford, the Chan Zuckerberg Initiative, the Kusuma Trust, and Novartis. He served on the data safety and monitoring board for trials of low‐dose interleukin‐2 therapy (IVORY and LILACS): sponsor University of Cambridge. There are no stock/equity/patent declarations for R.P.C. or K.B. or their immediate families.

## Data Availability

The authors have nothing to report.

## References

[1] G. Liuzzo , L. M. Biasucci , J. R. Gallimore , et al., “The Prognostic Value of C‐Reactive Protein and Serum Amyloid a Protein in Severe Unstable Angina,” New England Journal of Medicine 331 (1994): 417–424. 7880233 10.1056/NEJM199408183310701

[2] P. M. Ridker , M. Cushman , M. J. Stampfer , R. P. Tracy , and C. H. Hennekens , “Inflammation, Aspirin, and the Risk of Cardiovascular Disease in Apparently Healthy Men,” New England Journal of Medicine 336 (1997): 973–979. 9077376 10.1056/NEJM199704033361401

[3] P. M. Ridker , D. L. Bhatt , A. D. Pradhan , et al., “Inflammation and Cholesterol as Predictors of Cardiovascular Events Among Patients Receiving Statin Therapy: A Collaborative Analysis of Three Randomised Trials,” Lancet 401 (2023): 1293–1301. 36893777 10.1016/S0140-6736(23)00215-5

[4] H. Emami , P. Singh , M. MacNabb , et al., “Splenic Metabolic Activity Predicts Risk of Future Cardiovascular Events: Demonstration of a Cardiosplenic Axis in Humans,” JACC: Cardiovascular Imaging 8 (2015): 121–130. 25577441 10.1016/j.jcmg.2014.10.009 PMC6855915

[5] P. Elliott , J. C. Chambers , W. Zhang , et al., “Genetic Loci Associated With C‐Reactive Protein Levels and Risk of Coronary Heart Disease,” JAMA 302 (2009): 37–48. 19567438 10.1001/jama.2009.954 PMC2803020

[6] M. G. Netea , L. A. Joosten , E. Latz , et al., “Trained Immunity: A Program of Innate Immune Memory in Health and Disease,” Science 352 (2016): aaf1098. 27102489 10.1126/science.aaf1098 PMC5087274

[7] S. Bekkering , I. van den Munckhof , T. Nielen , et al., “Innate Immune Cell Activation and Epigenetic Remodeling in Symptomatic and Asymptomatic Atherosclerosis in Humans In Vivo,” Atherosclerosis 254 (2016): 228–236. 27764724 10.1016/j.atherosclerosis.2016.10.019

[8] J. Q. Mol , J. van Tuijl , S. Bekkering , et al., “Peripheral Blood Mononuclear Cell Hyperresponsiveness in Patients With Premature Myocardial Infarction Without Traditional Risk Factors,” iScience 26 (2023): 107183. 37456854 10.1016/j.isci.2023.107183 PMC10338301

[9] J. Zhang , F. Yang , Y. Liao , et al., “Enhanced Trained Immunity in Peripheral Monocytes in Unstable Angina With Elevated High‐Sensitivity C‐Reactive Protein,” JACC Basic Transl Sci 10 (2025): 101300. 40561640 10.1016/j.jacbts.2025.04.014 PMC12256326

[10] M. G. Netea , J. Quintin , and J. W. van der Meer , “Trained Immunity: A Memory for Innate Host Defense,” Cell Host & Microbe 9 (2011): 355–361. 21575907 10.1016/j.chom.2011.04.006

[11] J. Kleinnijenhuis , J. Quintin , F. Preijers , et al., “Bacille Calmette‐Guerin Induces NOD2‐Dependent Nonspecific Protection From Reinfection via Epigenetic Reprogramming of Monocytes,” Proceedings of the National Academy of Sciences of the United States of America 109 (2012): 17537–17542. 22988082 10.1073/pnas.1202870109 PMC3491454

[12] S. C. Cheng , J. Quintin , R. A. Cramer , et al., “mTOR‐ and HIF‐1α‐Mediated Aerobic Glycolysis as Metabolic Basis for Trained Immunity,” Science 345 (2014): 1250684. 25258083 10.1126/science.1250684 PMC4226238

[13] L. Edgar , N. Akbar , A. T. Braithwaite , et al., “Hyperglycemia Induces Trained Immunity in Macrophages and Their Precursors and Promotes Atherosclerosis,” Circulation 144 (2021): 961–982. 34255973 10.1161/CIRCULATIONAHA.120.046464 PMC8448412

[14] S. T. Keating , L. Groh , K. Thiem , et al., “Rewiring of Glucose Metabolism Defines Trained Immunity Induced by Oxidized Low‐Density Lipoprotein,” Journal of Molecular Medicine (Berlin) 98 (2020): 819–831. 10.1007/s00109-020-01915-w PMC7297856 32350546

[15] S. Bekkering , R. J. W. Arts , B. Novakovic , et al., “Metabolic Induction of Trained Immunity Through the Mevalonate Pathway,” Cell 172 (2018): 135–146.e9. 29328908 10.1016/j.cell.2017.11.025

[16] H. Y. Kim , Y. J. Kang , D. H. Kim , et al., “Uremic Toxin Indoxyl Sulfate Induces Trained Immunity via the AhR‐Dependent Arachidonic Acid Pathway in End‐Stage Renal Disease (ESRD),” eLife 12 (2024): 12. 10.7554/eLife.87316 PMC11233136 38980302

[17] H. Cai , X. Chen , Y. Liu , et al., “Lactate Activates Trained Immunity by Fueling the Tricarboxylic Acid Cycle and Regulating Histone Lactylation,” Nature Communications 16 (2025): 3230. 10.1038/s41467-025-58563-2 PMC11971257 40185732

[18] R. J. Arts , B. Novakovic , R. Ter Horst , et al., “Glutaminolysis and Fumarate Accumulation Integrate Immunometabolic and Epigenetic Programs in Trained Immunity,” Cell Metabolism 24 (2016): 807–819. 27866838 10.1016/j.cmet.2016.10.008 PMC5742541

[19] A. Scarpa , Y. Jung , A. Hamid , et al., “Trained Immunity Induced by Oxidized Low‐Density Lipoprotein Is Dependent on Glutaminolysis,” FASEB Journal 39 (2025): e70774. 40577101 10.1096/fj.202500802R PMC12204306

[20] P. Koivunen , M. Hirsilä , A. M. Remes , I. E. Hassinen , K. I. Kivirikko , and J. Myllyharju , “Inhibition of Hypoxia‐Inducible Factor (HIF) Hydroxylases by Citric Acid Cycle Intermediates: Possible Links Between Cell Metabolism and Stabilization of HIF,” Journal of Biological Chemistry 282 (2007): 4524–4532. 17182618 10.1074/jbc.M610415200

[21] L. M. Merlo Pich , A. Ziogas , A. V. Ferreira , et al., “Arginine Metabolism Supports Metabolic Reprogramming in Trained Immunity,” Journal of Leukocyte Biology 117 (2025): qiaf080. 40464645 10.1093/jleuko/qiaf080

[22] N. T. Crump , A. V. Hadjinicolaou , M. Xia , et al., “Chromatin Accessibility Governs the Differential Response of Cancer and T Cells to Arginine Starvation,” Cell Reports 35 (2021): 109101. 33979616 10.1016/j.celrep.2021.109101 PMC8131582

[23] K. Thiem , S. T. Keating , M. G. Netea , et al., “Hyperglycemic Memory of Innate Immune Cells Promotes In Vitro Proinflammatory Responses of Human Monocytes and Murine Macrophages,” Journal of Immunology 206 (2021): 807–813. 10.4049/jimmunol.1901348 33431659

[24] S. Bekkering , J. Quintin , L. A. Joosten , J. W. van der Meer , M. G. Netea , and N. P. Riksen , “Oxidized Low‐Density Lipoprotein Induces Long‐Term Proinflammatory Cytokine Production and Foam Cell Formation via Epigenetic Reprogramming of Monocytes,” Arteriosclerosis, Thrombosis, and Vascular Biology 34 (2014): 1731–1738. 24903093 10.1161/ATVBAHA.114.303887

[25] J. Quintin , S. Saeed , J. H. A. Martens , et al., “ *Candida albicans* Infection Affords Protection Against Reinfection via Functional Reprogramming of Monocytes,” Cell Host & Microbe 12 (2012): 223–232. 22901542 10.1016/j.chom.2012.06.006 PMC3864037

[26] S. J. C. F. Moorlag , N. Khan , B. Novakovic , et al., “β‐Glucan Induces Protective Trained Immunity Against *Mycobacterium tuberculosis* Infection: A Key Role for IL‐1,” Cell Reports 31 (2020): 107634. 32433977 10.1016/j.celrep.2020.107634 PMC7242907

[27] S. Saeed , J. Quintin , H. H. Kerstens , et al., “Epigenetic Programming of Monocyte‐To‐Macrophage Differentiation and Trained Innate Immunity,” Science 345 (2014): 1251086. 25258085 10.1126/science.1251086 PMC4242194

[28] S. T. Keating , L. Groh , C. D. C. C. van der Heijden , et al., “The Set7 Lysine Methyltransferase Regulates Plasticity in Oxidative Phosphorylation Necessary for Trained Immunity Induced by β‐Glucan,” Cell Reports 31 (2020): 107548. 32320649 10.1016/j.celrep.2020.107548 PMC7184679

[29] A. V. Ferreira , S. Kostidis , L. A. Groh , et al., “Dimethyl Itaconate Induces Long‐Term Innate Immune Responses and Confers Protection Against Infection,” Cell Reports 42 (2023): 112658. 37330914 10.1016/j.celrep.2023.112658

[30] J. C. Dos Santos , M. Moreno , L. U. Teufel , et al., “Leishmania Braziliensis Enhances Monocyte Responses to Promote Anti‐Tumor Activity,” Cell Reports 43 (2024): 113932. 38457336 10.1016/j.celrep.2024.113932 PMC11000460

[31] S. Bannister , B. Kim , J. Domínguez‐Andrés , et al., “Neonatal BCG Vaccination Is Associated With a Long‐Term DNA Methylation Signature in Circulating Monocytes,” Science Advances 8 (2022): eabn4002. 35930640 10.1126/sciadv.abn4002 PMC9355358

[32] A. Ziogas , B. Novakovic , L. Ventriglia , et al., “Long‐Term Histone Lactylation Connects Metabolic and Epigenetic Rewiring in Innate Immune Memory,” Cell 188 (2025): 2992–3012.e16. 40318634 10.1016/j.cell.2025.03.048

[33] D. Zhang , Z. Tang , H. Huang , et al., “Metabolic Regulation of Gene Expression by Histone Lactylation,” Nature 574 (2019): 575–580. 31645732 10.1038/s41586-019-1678-1 PMC6818755

[34] D. P. Schrijver , R. J. Röring , J. Deckers , et al., “Resolving Sepsis‐Induced Immunoparalysis via Trained Immunity by Targeting Interleukin‐4 to Myeloid Cells,” Nature Biomedical Engineering 7 (2023): 1097–1112. 10.1038/s41551-023-01050-0 PMC10504080 37291433

[35] V. P. Mourits , J. H. van Puffelen , B. Novakovic , et al., “Lysine Methyltransferase G9a Is an Important Modulator of Trained Immunity,” Clinical & Translational Immunology 10 (2021): e1253. 33708384 10.1002/cti2.1253 PMC7890679

[36] S. J. C. F. Moorlag , V. Matzaraki , J. H. van Puffelen , et al., “An Integrative Genomics Approach Identifies KDM4 as a Modulator of Trained Immunity,” European Journal of Immunology 52 (2022): 431–446. 34821391 10.1002/eji.202149577 PMC9299854

[37] B. Novakovic , E. Habibi , S. Y. Wang , et al., “β‐Glucan Reverses the Epigenetic State of LPS‐Induced Immunological Tolerance,” Cell 167 (2016): 1354–1368.e14. 27863248 10.1016/j.cell.2016.09.034 PMC5927328

[38] L. Kruidenier , C. W. Chung , Z. Cheng , et al., “A Selective Jumonji H3K27 Demethylase Inhibitor Modulates the Proinflammatory Macrophage Response,” Nature 488 (2012): 404–408. 22842901 10.1038/nature11262 PMC4691848

[39] S. Fanucchi , E. T. Fok , E. Dalla , et al., “Immune Genes Are Primed for Robust Transcription by Proximal Long Noncoding RNAs Located in Nuclear Compartments,” Nature Genetics 51 (2019): 138–150. 30531872 10.1038/s41588-018-0298-2

[40] E. T. Fok , S. J. C. F. Moorlag , Y. Negishi , et al., “A Chromatin‐Regulated Biphasic Circuit Coordinates IL‐1β‐Mediated Inflammation,” Nature Genetics 56 (2024): 85–99. 38092881 10.1038/s41588-023-01598-2

[41] G. Cavalli , I. W. Tengesdal , M. Gresnigt , et al., “The Anti‐Inflammatory Cytokine Interleukin‐37 Is an Inhibitor of Trained Immunity,” Cell Reports 35 (2021): 108955. 33826894 10.1016/j.celrep.2021.108955

[42] I. Mitroulis , K. Ruppova , B. Wang , et al., “Modulation of Myelopoiesis Progenitors Is an Integral Component of Trained Immunity,” Cell 172 (2018): 147–161.e12. 29328910 10.1016/j.cell.2017.11.034 PMC5766828

[43] I. Mitroulis , G. Hajishengallis , and T. Chavakis , “Trained Immunity and Cardiometabolic Disease: The Role of Bone Marrow,” Arteriosclerosis, Thrombosis, and Vascular Biology 41 (2021): 48–54. 33207931 10.1161/ATVBAHA.120.314215 PMC7769996

[44] S. Bekkering , C. Saner , B. Novakovic , et al., “Increased Innate Immune Responses in Adolescents With Obesity and Its Relation to Subclinical Cardiovascular Measures: An Exploratory Study,” iScience 27 (2024): 109762. 38741712 10.1016/j.isci.2024.109762 PMC11089376

[45] Z. Dong , L. Hou , W. Luo , et al., “Myocardial Infarction Drives Trained Immunity of Monocytes, Accelerating Atherosclerosis,” European Heart Journal 45 (2024): 669–684. 38085922 10.1093/eurheartj/ehad787

[46] E. Jentho , C. Ruiz‐Moreno , B. Novakovic , et al., “Trained Innate Immunity, Long‐Lasting Epigenetic Modulation, and Skewed Myelopoiesis by Heme,” Proceedings of the National Academy of Sciences of the United States of America 118 (2021): e2102698118. 34663697 10.1073/pnas.2102698118 PMC8545490

[47] A. M. Rehill , S. McCluskey , A. E. Ledwith , et al., “Trained Immunity Causes Myeloid Cell Hypercoagulability,” Science Advances 11 (2025): eads0105. 40053582 10.1126/sciadv.ads0105 PMC11887800

[48] I. M. Stratton , A. I. Adler , H. A. Neil , et al., “Association of Glycaemia With Macrovascular and Microvascular Complications of Type 2 Diabetes (UKPDS 35): Prospective Observational Study,” BMJ 321 (2000): 405–412. 10938048 10.1136/bmj.321.7258.405 PMC27454

[49] T. T. J. Kufazvinei , J. Chai , K. A. Boden , K. M. Channon , and R. P. Choudhury , “Emerging Opportunities to Target Inflammation: Myocardial Infarction and Type 2 Diabetes,” Cardiovascular Research 120 (2024): 1241–1252. 39027945 10.1093/cvr/cvae142 PMC11416061

[50] J. D. Newman , A. K. Vani , J. O. Aleman , H. S. Weintraub , J. S. Berger , and A. Z. Schwartzbard , “The Changing Landscape of Diabetes Therapy for Cardiovascular Risk Reduction: JACC State‐Of‐The‐Art Review,” Journal of the American College of Cardiology 72 (2018): 1856–1869. 30286929 10.1016/j.jacc.2018.07.071 PMC6178226

[51] H. C. Gerstein , M. E. Miller , S. Genuth , et al., “Long‐Term Effects of Intensive Glucose Lowering on Cardiovascular Outcomes,” New England Journal of Medicine 364 (2011): 818–828. 21366473 10.1056/NEJMoa1006524 PMC4083508

[52] B. Zinman , C. Wanner , J. M. Lachin , et al., “Empagliflozin, Cardiovascular Outcomes, and Mortality in Type 2 Diabetes,” New England Journal of Medicine 373 (2015): 2117–2128. 26378978 10.1056/NEJMoa1504720

[53] R. R. Holman , S. K. Paul , M. A. Bethel , D. R. Matthews , and H. A. Neil , “10‐Year Follow‐Up of Intensive Glucose Control in Type 2 Diabetes,” New England Journal of Medicine 359 (2008): 1577–1589. 18784090 10.1056/NEJMoa0806470

[54] “Intensive Blood‐Glucose Control With Sulphonylureas or Insulin Compared With Conventional Treatment and Risk of Complications in Patients With Type 2 Diabetes (UKPDS 33),” UK Prospective Diabetes Study (UKPDS) Group Lancet 352 (1998): 837–853. 9742976

[55] D. M. Nathan , S. Genuth , J. Lachin , et al., “The Effect of Intensive Treatment of Diabetes on the Development and Progression of Long‐Term Complications in Insulin‐Dependent Diabetes Mellitus,” New England Journal of Medicine 329 (1993): 977–986. 8366922 10.1056/NEJM199309303291401

[56] D. M. Nathan , P. A. Cleary , J. Y. Backlund , et al., “Intensive Diabetes Treatment and Cardiovascular Disease in Patients With Type 1 Diabetes,” New England Journal of Medicine 353 (2005): 2643–2653. 16371630 10.1056/NEJMoa052187 PMC2637991

[57] A. I. Adler , R. L. Coleman , J. Leal , W. N. Whiteley , P. Clarke , and R. R. Holman , “Post‐Trial Monitoring of a Randomised Controlled Trial of Intensive Glycaemic Control in Type 2 Diabetes Extended From 10 Years to 24 Years (UKPDS 91),” Lancet 404 (2024): 145–155. 38772405 10.1016/S0140-6736(24)00537-3

[58] E. Gkrania‐Klotsas , Z. Ye , A. J. Cooper , et al., “Differential White Blood Cell Count and Type 2 Diabetes: Systematic Review and Meta‐Analysis of Cross‐Sectional and Prospective Studies,” PLoS One 5 (2010): e13405. 20976133 10.1371/journal.pone.0013405 PMC2956635

[59] F. F. Hoyer , X. Zhang , E. Coppin , et al., “Bone Marrow Endothelial Cells Regulate Myelopoiesis in Diabetes Mellitus,” Circulation 142 (2020): 244–258. 32316750 10.1161/CIRCULATIONAHA.120.046038 PMC7375017

[60] P. R. Nagareddy , A. J. Murphy , R. A. Stirzaker , et al., “Hyperglycemia Promotes Myelopoiesis and Impairs the Resolution of Atherosclerosis,” Cell Metabolism 17 (2013): 695–708. 23663738 10.1016/j.cmet.2013.04.001 PMC3992275

[61] T. J. Barrett , A. J. Murphy , I. J. Goldberg , and E. A. Fisher , “Diabetes‐Mediated Myelopoiesis and the Relationship to Cardiovascular Risk,” Annals of the New York Academy of Sciences 1402 (2017): 31–42. 28926114 10.1111/nyas.13462 PMC5659728

[62] M. C. Flynn , M. J. Kraakman , C. Tikellis , et al., “Transient Intermittent Hyperglycemia Accelerates Atherosclerosis by Promoting Myelopoiesis,” Circulation Research 127 (2020): 877–892. 32564710 10.1161/CIRCRESAHA.120.316653 PMC7486277

[63] X. He , X. Wang , J. van Heck , et al., “Blood Immune Cell Profiling in Adults With Longstanding Type 1 Diabetes Is Associated With Macrovascular Complications,” Frontiers in Immunology 15 (2024): 1401542. 39011037 10.3389/fimmu.2024.1401542 PMC11246869

[64] Y. D'Alessandra , M. Chiesa , V. Vigorelli , et al., “Diabetes Induces a Transcriptional Signature in Bone Marrow‐Derived CD34,” International Journal of Molecular Sciences 22 (2021): 1423. 33572602 10.3390/ijms22031423 PMC7866997

[65] M. C. Vinci , S. Costantino , G. Damiano , et al., “Persistent Epigenetic Signals Propel a Senescence‐Associated Secretory Phenotype and Trained Innate Immunity in CD34,” Cardiovascular Diabetology 23 (2024): 107. 38553774 10.1186/s12933-024-02195-1 PMC10981360

[66] M. G. Netea , J. Domínguez‐Andrés , L. B. Barreiro , et al., “Defining Trained Immunity and Its Role in Health and Disease,” Nature Reviews. Immunology 20 (2020): 375–388. 10.1038/s41577-020-0285-6 PMC7186935 32132681

[67] F. Miao , X. Wu , L. Zhang , Y. C. Yuan , A. D. Riggs , and R. Natarajan , “Genome‐Wide Analysis of Histone Lysine Methylation Variations Caused by Diabetic Conditions in Human Monocytes,” Journal of Biological Chemistry 282 (2007): 13854–13863. 17339327 10.1074/jbc.M609446200

[68] F. Miao , I. G. Gonzalo , L. Lanting , and R. Natarajan , “In Vivo Chromatin Remodeling Events Leading to Inflammatory Gene Transcription Under Diabetic Conditions,” Journal of Biological Chemistry 279 (2004): 18091–18097. 14976218 10.1074/jbc.M311786200

[69] C. N. Lumeng , J. L. Bodzin , and A. R. Saltiel , “Obesity Induces a Phenotypic Switch in Adipose Tissue Macrophage Polarization,” Journal of Clinical Investigation 117 (2007): 175–184. 17200717 10.1172/JCI29881 PMC1716210

[70] A. M. Blaszczak , M. Bernier , V. P. Wright , et al., “Obesogenic Memory Maintains Adipose Tissue Inflammation and Insulin Resistance,” Immunometabolism 2 (2020): 2. 10.20900/immunometab20200023 PMC7409818 32774894

[71] J. van Tuijl , D. Vreeken , W. Broeders , et al., “The Long‐Term Effect of Metabolic Bariatric Surgery on Innate Immune Cell Phenotype and Function,” International Journal of Obesity (London, England) 49 (2025): 2473–2483. 10.1038/s41366-025-01886-3 40836110

[72] H. L. Caslin , M. A. Cottam , J. M. Piñon , L. Y. Boney , and A. H. Hasty , “Weight Cycling Induces Innate Immune Memory in Adipose Tissue Macrophages,” Frontiers in Immunology 13 (2022): 984859. 36713396 10.3389/fimmu.2022.984859 PMC9876596

[73] M. A. Cottam , H. L. Caslin , N. C. Winn , and A. H. Hasty , “Multiomics Reveals Persistence of Obesity‐Associated Immune Cell Phenotypes in Adipose Tissue During Weight Loss and Weight Regain in Mice,” Nature Communications 13 (2022): 2950. 10.1038/s41467-022-30646-4 PMC9135744 35618862

[74] R. Ter Horst , I. C. L. van den Munckhof , K. Schraa , et al., “Sex‐Specific Regulation of Inflammation and Metabolic Syndrome in Obesity,” Arteriosclerosis, Thrombosis, and Vascular Biology 40 (2020): 1787–1800. 32460579 10.1161/ATVBAHA.120.314508 PMC7310302

[75] D. Flores Gomez , S. Bekkering , R. Ter Horst , et al., “The Effect of Leptin on Trained Innate Immunity and on Systemic Inflammation in Subjects With Obesity,” Journal of Leukocyte Biology 115 (2024): 374–384. 37776323 10.1093/jleuko/qiad118

[76] K. Singer , J. DelProposto , D. L. Morris , et al., “Diet‐Induced Obesity Promotes Myelopoiesis in Hematopoietic Stem Cells,” Molecular Metabolism 3 (2014): 664–675. 25161889 10.1016/j.molmet.2014.06.005 PMC4142398

[77] P. R. Nagareddy , M. Kraakman , S. L. Masters , et al., “Adipose Tissue Macrophages Promote Myelopoiesis and Monocytosis in Obesity,” Cell Metabolism 19 (2014): 821–835. 24807222 10.1016/j.cmet.2014.03.029 PMC4048939

[78] S. Bekkering , L. C. A. Stiekema , S. Bernelot Moens , et al., “Treatment With Statins Does Not Revert Trained Immunity in Patients With Familial Hypercholesterolemia,” Cell Metabolism 30 (2019): 1–2. 31204280 10.1016/j.cmet.2019.05.014

[79] L. C. A. Stiekema , L. Willemsen , Y. Kaiser , et al., “Impact of Cholesterol on Proinflammatory Monocyte Production by the Bone Marrow,” European Heart Journal 42 (2021): 4309–4320. 34343254 10.1093/eurheartj/ehab465 PMC8572558

[80] T. Seijkens , M. A. Hoeksema , L. Beckers , et al., “Hypercholesterolemia‐Induced Priming of Hematopoietic Stem and Progenitor Cells Aggravates Atherosclerosis,” FASEB Journal 28 (2014): 2202–2213. 24481967 10.1096/fj.13-243105

[81] L. A. Groh , A. V. Ferreira , L. Helder , et al., “oxLDL‐Induced Trained Immunity Is Dependent on Mitochondrial Metabolic Reprogramming,” Immunometabolism 3 (2021): e210025. 34267957 10.20900/immunometab20210025 PMC7611242

[82] Y. Wang , G. Z. Wang , P. S. Rabinovitch , and I. Tabas , “Macrophage Mitochondrial Oxidative Stress Promotes Atherosclerosis and Nuclear Factor‐κB‐Mediated Inflammation in Macrophages,” Circulation Research 114 (2014): 421–433. 24297735 10.1161/CIRCRESAHA.114.302153 PMC3946745

[83] F. M. van der Valk , S. Bekkering , J. Kroon , et al., “Oxidized Phospholipids on Lipoprotein(a) Elicit Arterial Wall Inflammation and an Inflammatory Monocyte Response in Humans,” Circulation 134 (2016): 611–624. 27496857 10.1161/CIRCULATIONAHA.116.020838 PMC4995139

[84] A. Christ , M. Lauterbach , and E. Latz , “Western Diet and the Immune System: An Inflammatory Connection,” Immunity 51 (2019): 794–811. 31747581 10.1016/j.immuni.2019.09.020

[85] A. Christ , P. Günther , M. A. R. Lauterbach , et al., “Western Diet Triggers NLRP3‐Dependent Innate Immune Reprogramming,” Cell 172 (2018): 162–175.e14. 29328911 10.1016/j.cell.2017.12.013 PMC6324559

[86] E. van Kampen , A. Jaminon , T. J. van Berkel , and M. Van Eck , “Diet‐Induced (Epigenetic) Changes in Bone Marrow Augment Atherosclerosis,” Journal of Leukocyte Biology 96 (2014): 833–841. 25024399 10.1189/jlb.1A0114-017R

[87] P. K. Whelton , L. J. Appel , R. L. Sacco , et al., “Sodium, Blood Pressure, and Cardiovascular Disease: Further Evidence Supporting the American Heart Association Sodium Reduction Recommendations,” Circulation 126 (2012): 2880–2889. 23124030 10.1161/CIR.0b013e318279acbf

[88] T. Y. Lin , D. Jiang , W. R. Chen , et al., “Trained Immunity Induced by High‐Salt Diet Impedes Stroke Recovery,” EMBO Reports 24 (2023): e57164. 37965920 10.15252/embr.202357164 PMC10702837

[89] H. Chen , J. Song , L. Zeng , et al., “Dietary Sodium Modulates mTORC1‐Dependent Trained Immunity in Macrophages to Accelerate CKD Development,” Biochemical Pharmacology 229 (2024): 116505. 39181336 10.1016/j.bcp.2024.116505

[90] J. E. Dimsdale , “Psychological Stress and Cardiovascular Disease,” Journal of the American College of Cardiology 51 (2008): 1237–1246. 18371552 10.1016/j.jacc.2007.12.024 PMC2633295

[91] M. Esler , “Mental Stress and Human Cardiovascular Disease,” Neuroscience and Biobehavioral Reviews 74 (2017): 269–276. 27751732 10.1016/j.neubiorev.2016.10.011

[92] A. Rosengren , S. Hawken , S. Ounpuu , et al., “Association of Psychosocial Risk Factors With Risk of Acute Myocardial Infarction in 11119 Cases and 13648 Controls From 52 Countries (The INTERHEART Study): Case‐Control Study,” Lancet 364 (2004): 953–962. 15364186 10.1016/S0140-6736(04)17019-0

[93] S. V. Arnold , K. G. Smolderen , D. M. Buchanan , Y. Li , and J. A. Spertus , “Perceived Stress in Myocardial Infarction: Long‐Term Mortality and Health Status Outcomes,” Journal of the American College of Cardiology 60 (2012): 1756–1763. 23040574 10.1016/j.jacc.2012.06.044 PMC3601381

[94] T. Heidt , H. B. Sager , G. Courties , et al., “Chronic Variable Stress Activates Hematopoietic Stem Cells,” Nature Medicine 20 (2014): 754–758. 10.1038/nm.3589 PMC4087061 24952646

[95] P. Dutta , G. Courties , Y. Wei , et al., “Myocardial Infarction Accelerates Atherosclerosis,” Nature 487 (2012): 325–329. 22763456 10.1038/nature11260 PMC3401326

[96] C. D. C. C. van der Heijden , L. Groh , S. T. Keating , et al., “Catecholamines Induce Trained Immunity in Monocytes In Vitro and In Vivo,” Circulation Research 127 (2020): 269–283. 32241223 10.1161/CIRCRESAHA.119.315800

[97] T. J. Barrett , E. M. Corr , C. van Solingen , et al., “Chronic Stress Primes Innate Immune Responses in Mice and Humans,” Cell Reports 36 (2021): 109595. 34496250 10.1016/j.celrep.2021.109595 PMC8493594

[98] M. Ntsekhe and J. V. Baker , “Cardiovascular Disease Among Persons Living With HIV: New Insights Into Pathogenesis and Clinical Manifestations in a Global Context,” Circulation 147 (2023): 83–100. 36576956 10.1161/CIRCULATIONAHA.122.057443

[99] A. G. Vos , N. S. Idris , R. E. Barth , K. Klipstein‐Grobusch , and D. E. Grobbee , “Pro‐Inflammatory Markers in Relation to Cardiovascular Disease in HIV Infection. A Systematic Review,” PLoS One 11 (2016): e0147484. 26808540 10.1371/journal.pone.0147484 PMC4726827

[100] D. B. Hanna , J. Lin , W. S. Post , et al., “Association of Macrophage Inflammation Biomarkers With Progression of Subclinical Carotid Artery Atherosclerosis in HIV‐Infected Women and Men,” Journal of Infectious Diseases 215 (2017): 1352–1361. 28199691 10.1093/infdis/jix082 PMC5722037

[101] W. A. van der Heijden , L. Van de Wijer , F. Keramati , et al., “Chronic HIV Infection Induces Transcriptional and Functional Reprogramming of Innate Immune Cells,” JCI Insight 6 (2021): 6. 10.1172/jci.insight.145928 PMC8119206 33630761

[102] L. Dubrovsky , B. Brichacek , N. M. Prashant , et al., “Extracellular Vesicles Carrying HIV‐1 Nef Induce Long‐Term Hyperreactivity of Myeloid Cells,” Cell Reports 41 (2022): 111674. 36417867 10.1016/j.celrep.2022.111674 PMC9733434

[103] E. Bartoloni , A. Alunno , G. Cafaro , et al., “Subclinical Atherosclerosis in Primary Sjögren's Syndrome: Does Inflammation Matter?,” Frontiers in Immunology 10 (2019): 817. 31110500 10.3389/fimmu.2019.00817 PMC6499202

[104] E. Bartoloni , C. Baldini , G. Schillaci , et al., “Cardiovascular Disease Risk Burden in Primary Sjögren's Syndrome: Results of a Population‐Based Multicentre Cohort Study,” Journal of Internal Medicine 278 (2015): 185–192. 25582881 10.1111/joim.12346

[105] R. Tobin , N. Patel , K. Tobb , B. Weber , P. K. Mehta , and I. Isiadinso , “Atherosclerosis in Systemic Lupus Erythematosus,” Current Atherosclerosis Reports 25 (2023): 819–827. 37768411 10.1007/s11883-023-01149-4

[106] P. M. Ridker , “Autoimmune Diseases and Atherothrombotic Risk,” Lancet 400 (2022): 708–710. 36041476 10.1016/S0140-6736(22)01602-6

[107] E. Huijser , C. G. van Helden‐Meeuwsen , D. G. B. Grashof , et al., “Trained Immunity in Primary Sjögren's Syndrome: Linking Type I Interferons to a Pro‐Atherogenic Phenotype,” Frontiers in Immunology 13 (2022): 840751. 35860283 10.3389/fimmu.2022.840751 PMC9289449

[108] C. Yanginlar , N. Rother , T. G. J. M. Post , et al., “Trained Innate Immunity in Response to Nuclear Antigens in Systemic Lupus Erythematosus,” Journal of Autoimmunity 149 (2024): 103335. 39549487 10.1016/j.jaut.2024.103335

[109] T. McGarry , M. M. Hanlon , V. Marzaioli , et al., “Rheumatoid Arthritis CD14,” Clinical & Translational Immunology 10 (2021): e1237. 33510894 10.1002/cti2.1237 PMC7815439

[110] L. D. Ferguson , G. Molenberghs , G. Verbeke , et al., “Gout and Incidence of 12 Cardiovascular Diseases: A Case‐Control Study Including 152 663 Individuals With Gout and 709 981 Matched Controls,” Lancet Rheumatology 6 (2024): e156–e167. 38383089 10.1016/S2665-9913(23)00338-7

[111] T. O. Crişan , M. C. P. Cleophas , B. Novakovic , et al., “Uric Acid Priming in Human Monocytes Is Driven by the AKT‐PRAS40 Autophagy Pathway,” Proceedings of the National Academy of Sciences of the United States of America 114 (2017): 5485–5490. 28484006 10.1073/pnas.1620910114 PMC5448210

[112] G. Hajishengallis and T. Chavakis , “Local and Systemic Mechanisms Linking Periodontal Disease and Inflammatory Comorbidities,” Nature Reviews. Immunology 21 (2021): 426–440. 10.1038/s41577-020-00488-6 PMC7841384 33510490

[113] X. Li , H. Wang , X. Yu , et al., “Maladaptive Innate Immune Training of Myelopoiesis Links Inflammatory Comorbidities,” Cell 185 (2022): 1709–1727.e18. 35483374 10.1016/j.cell.2022.03.043 PMC9106933

[114] M. P. Noz , A. S. Plachokova , E. M. M. Smeets , et al., “An Explorative Study on Monocyte Reprogramming in the Context of Periodontitis,” Frontiers in Immunology 12 (2021): 695227. 34484192 10.3389/fimmu.2021.695227 PMC8414567

[115] S. Kato , M. Chmielewski , H. Honda , et al., “Aspects of Immune Dysfunction in End‐Stage Renal Disease,” Clinical Journal of the American Society of Nephrology 3 (2008): 1526–1533. 18701615 10.2215/CJN.00950208 PMC4571158

[116] R. Vanholder , G. Glorieux , N. Lameire , and Group EUTW , “Uraemic Toxins and Cardiovascular Disease,” Nephrology, Dialysis, Transplantation 18 (2003): 463–466. 10.1093/ndt/18.3.463 12584262

[117] H. Gao and S. Liu , “Role of Uremic Toxin Indoxyl Sulfate in the Progression of Cardiovascular Disease,” Life Sciences 185 (2017): 23–29. 28754616 10.1016/j.lfs.2017.07.027

[118] G. Chen , H. Chen , S. Ren , et al., “Aberrant DNA Methylation of mTOR Pathway Genes Promotes Inflammatory Activation of Immune Cells in Diabetic Kidney Disease,” Kidney International 96 (2019): 409–420. 31101365 10.1016/j.kint.2019.02.020

[119] I. Jonkman , M. M. E. Jacobs , Y. Negishi , et al., “Trained Immunity Suppression Determines Kidney Allograft Survival,” American Journal of Transplantation 24 (2024): 2022–2033. 39147201 10.1016/j.ajt.2024.08.006 PMC11789421

[120] J. Rangaswami , R. O. Mathew , R. Parasuraman , et al., “Cardiovascular Disease in the Kidney Transplant Recipient: Epidemiology, Diagnosis and Management Strategies,” Nephrology, Dialysis, Transplantation 34 (2019): 760–773. 10.1093/ndt/gfz053 30984976

[121] S. Monticone , F. D'Ascenzo , C. Moretti , et al., “Cardiovascular Events and Target Organ Damage in Primary Aldosteronism Compared With Essential Hypertension: A Systematic Review and Meta‐Analysis,” Lancet Diabetes and Endocrinology 6 (2018): 41–50. 29129575 10.1016/S2213-8587(17)30319-4

[122] A. P. McGraw , J. Bagley , W. S. Chen , et al., “Aldosterone Increases Early Atherosclerosis and Promotes Plaque Inflammation Through a Placental Growth Factor‐Dependent Mechanism,” Journal of the American Heart Association 2 (2013): e000018. 23525413 10.1161/JAHA.112.000018 PMC3603255

[123] L. A. P. Vilela and M. Q. Almeida , “Diagnosis and Management of Primary Aldosteronism,” Archives of Endocrinology and Metabolism 61 (2017): 305–312. 28699986 10.1590/2359-3997000000274 PMC10118808

[124] C. D. C. C. van der Heijden , S. T. Keating , L. Groh , L. A. B. Joosten , M. G. Netea , and N. P. Riksen , “Aldosterone Induces Trained Immunity: The Role of Fatty Acid Synthesis,” Cardiovascular Research 116 (2020): 317–328. 31119285 10.1093/cvr/cvz137

[125] Y. Xie , E. Xu , B. Bowe , and Z. Al‐Aly , “Long‐Term Cardiovascular Outcomes of COVID‐19,” Nature Medicine 28 (2022): 583–590. 10.1038/s41591-022-01689-3 PMC8938267 35132265

[126] M. Nishiga , D. W. Wang , Y. Han , D. B. Lewis , and J. C. Wu , “COVID‐19 and Cardiovascular Disease: From Basic Mechanisms to Clinical Perspectives,” Nature Reviews. Cardiology 17 (2020): 543–558. 32690910 10.1038/s41569-020-0413-9 PMC7370876

[127] N. Rother , C. Yanginlar , R. G. H. Lindeboom , et al., “Hydroxychloroquine Inhibits the Trained Innate Immune Response to Interferons,” Cell Reports Medicine 1 (2020): 100146. 33377122 10.1016/j.xcrm.2020.100146 PMC7762774

[128] J. Cvetkovic , R. H. J. Jacobi , A. Miranda‐Bedate , et al., “Human Monocytes Exposed to SARS‐CoV‐2 Display Features of Innate Immune Memory Producing High Levels of CXCL10 Upon Restimulation,” Journal of Innate Immunity 15 (2023): 911–924. 37989107 10.1159/000535120 PMC10718582

[129] S. J. Theobald , A. Simonis , T. Georgomanolis , et al., “Long‐Lived Macrophage Reprogramming Drives Spike Protein‐Mediated Inflammasome Activation in COVID‐19,” EMBO Molecular Medicine 13 (2021): e14150. 34133077 10.15252/emmm.202114150 PMC8350892

[130] P. M. Ridker , B. M. Everett , T. Thuren , et al., “Antiinflammatory Therapy With Canakinumab for Atherosclerotic Disease,” New England Journal of Medicine 377 (2017): 1119–1131. 28845751 10.1056/NEJMoa1707914

[131] P. M. Ridker , J. G. MacFadyen , B. M. Everett , et al., “Relationship of C‐Reactive Protein Reduction to Cardiovascular Event Reduction Following Treatment With Canakinumab: A Secondary Analysis From the CANTOS Randomised Controlled Trial,” Lancet 391 (2018): 319–328. 29146124 10.1016/S0140-6736(17)32814-3

[132] P. M. Ridker , P. Libby , J. G. MacFadyen , et al., “Modulation of the Interleukin‐6 Signalling Pathway and Incidence Rates of Atherosclerotic Events and All‐Cause Mortality: Analyses From the Canakinumab Anti‐Inflammatory Thrombosis Outcomes Study (CANTOS),” European Heart Journal 39 (2018): 3499–3507. 30165610 10.1093/eurheartj/ehy310

[133] P. M. Ridker , M. Devalaraja , F. M. M. Baeres , et al., “IL‐6 Inhibition With Ziltivekimab in Patients at High Atherosclerotic Risk (RESCUE): A Double‐Blind, Randomised, Placebo‐Controlled, Phase 2 Trial,” Lancet 397 (2021): 2060–2069. 34015342 10.1016/S0140-6736(21)00520-1

[134] L. Ulander , H. Tolppanen , O. Hartman , et al., “Hydroxychloroquine Reduces Interleukin‐6 Levels After Myocardial Infarction: The Randomized, Double‐Blind, Placebo‐Controlled OXI Pilot Trial,” International Journal of Cardiology 337 (2021): 21–27. 33961943 10.1016/j.ijcard.2021.04.062

[135] P. M. Ridker , B. M. Everett , A. Pradhan , et al., “Low‐Dose Methotrexate for the Prevention of Atherosclerotic Events,” New England Journal of Medicine 380 (2019): 752–762. 30415610 10.1056/NEJMoa1809798 PMC6587584

[136] J. C. Tardif , S. Kouz , D. D. Waters , et al., “Efficacy and Safety of Low‐Dose Colchicine After Myocardial Infarction,” New England Journal of Medicine 381 (2019): 2497–2505. 31733140 10.1056/NEJMoa1912388

[137] S. M. Nidorf , A. T. L. Fiolet , A. Mosterd , et al., “Colchicine in Patients With Chronic Coronary Disease,” New England Journal of Medicine 383 (2020): 1838–1847. 32865380 10.1056/NEJMoa2021372

[138] S. S. Jolly , M. A. d'Entremont , S. F. Lee , et al., “Colchicine in Acute Myocardial Infarction,” New England Journal of Medicine 392 (2025): 633–642. 39555823 10.1056/NEJMoa2405922

[139] R. Sriranjan , T. X. Zhao , J. Tarkin , et al., “Low‐Dose,” BMJ Open 12 (2022): e062602. 10.1136/bmjopen-2022-062602 PMC9558794 36207050

[140] T. X. Zhao , R. S. Sriranjan , Z. K. Tuong , et al., “Regulatory T‐Cell Response to Low‐Dose Interleukin‐2 in Ischemic Heart Disease,” NEJM Evidence 1 (2022): EVIDoa2100009. 38319239 10.1056/EVIDoa2100009

[141] A. Cordova Sanchez , F. Khokhar , D. A. Olonoff , and R. L. Carhart , “Hydroxychloroquine and Cardiovascular Events in Patients With Rheumatoid Arthritis,” Cardiovascular Drugs and Therapy 38 (2024): 297–304. 36197529 10.1007/s10557-022-07387-z PMC9532807

[142] J. H. Haugaard , L. Dreyer , M. B. Ottosen , G. Gislason , K. Kofoed , and A. Egeberg , “Use of Hydroxychloroquine and Risk of Major Adverse Cardiovascular Events in Patients With Lupus Erythematosus: A Danish Nationwide Cohort Study,” Journal of the American Academy of Dermatology 84 (2021): 930–937. 33321159 10.1016/j.jaad.2020.12.013

[143] M. R. Hoque , J. A. Aviña‐Zubieta , D. Lacaille , et al., “Antimalarial Adherence and Risk of Cardiovascular Events in Patients With Rheumatoid Arthritis and Systemic Lupus Erythematosus: A Population‐Based Study,” Arthritis Care & Research (Hoboken) 76 (2024): 426–436. 10.1002/acr.25233 37691305

[144] E. D'Andrea , R. J. Desai , M. He , et al., “Cardiovascular Risks of Hydroxychloroquine vs Methotrexate in Patients With Rheumatoid Arthritis,” Journal of the American College of Cardiology 80 (2022): 36–46. 35772915 10.1016/j.jacc.2022.04.039 PMC9722228

[145] Z. Mallat , A. Gojova , V. Brun , et al., “Induction of a Regulatory T Cell Type 1 Response Reduces the Development of Atherosclerosis in Apolipoprotein E‐Knockout Mice,” Circulation 108 (2003): 1232–1237. 12912803 10.1161/01.CIR.0000089083.61317.A1

[146] S. Kabacik , D. Lowe , L. Fransen , et al., “The Relationship Between Epigenetic Age and the Hallmarks of Aging in Human Cells,” Nature Aging 2 (2022): 484–493. 37034474 10.1038/s43587-022-00220-0 PMC10077971

